# Neonatal resuscitation practices in Uganda: a video observational study

**DOI:** 10.1136/bmjpo-2021-001092

**Published:** 2021-09-14

**Authors:** Daniel Helldén, Susanna Myrnerts Höök, Nicolas J Pejovic, Dan Mclellan, Clare Lubulwa, Thorkild Tylleskär, Tobias Alfven

**Affiliations:** 1 Global Public Health, Karolinska Institute, Stockholm, Sweden; 2 Center for International Health, University of Bergen Faculty of Medicine and Dentistry, Bergen, Norway; 3 Sachs’ Children and Youth Hospital, Stockholm, Sweden; 4 Mulago Specialized Women and Neonatal Hospital, Kampala, Uganda; 5 Centre for Intervention Science in Maternal and Child Health, Department of Global Public Health and Primary Care, University of Bergen, Bergen, Norway

**Keywords:** neonatology, resuscitation, health services research

## Abstract

**Background:**

Neonatal mortality, often due to birth asphyxia, remains stubbornly high in sub-Saharan Africa. Guidelines for neonatal resuscitation, where achieving adequate positive pressure ventilation (PPV) is key, have been implemented in low-resource settings. However, the actual clinical practices of neonatal resuscitation have rarely been examined in these settings. The primary aim of this prospective observational study was to detail the cumulative proportion of time with ventilation during the first minute on the resuscitation table of neonates needing PPV at the Mulago National Referral Hospital in Kampala, Uganda.

**Methods:**

From November 2015 to January 2016, resuscitations of non-breathing neonates by birth attendants were video-recorded using motion sensor cameras. The resuscitation practices were analysed using the application NeoTapAS and compared between those taking place in the labour ward and those in theatre through Fisher’s exact test and Wilcoxon rank-sum test.

**Results:**

From 141 recorded resuscitations, 99 were included for analysis. The time to initiation of PPV was 66 (42–102) s overall, and there was minimal PPV during the first minute in both groups with 0 (0–10) s and 0 (0–12) s of PPV, respectively. After initiating PPV the overall duration of interruptions during the first minute was 28 (18–37) s. Majority of interruptions were caused by stimulation (28%), unknown reasons (25%) and suction (22%).

**Conclusions:**

Our findings show a low adherence to standard resuscitation practices in 2015–2016. This emphasises the need for continuous educational efforts and investments in staff and adequate resources to increase the quality of clinical neonatal resuscitation practices in low-resource settings.

**What is known about the subject?**

- Timely initiation and duration of adequate ventilation is key to high-quality neonatal resuscitation.
- Educational programmes such as Helping Babies Breathe have been implemented to improve clinical practice in neonatal resuscitation.

**What this study adds?**

- Actual neonatal resuscitation practices still suffer from inadequate initiation and duration of positive pressure ventilation, tendency for overstimulation and excessive focus on suction.
- There is a strong need for continuous educational efforts and investments in staff and resources to increase the quality of neonatal resuscitation practices in low-resource settings.

## Introduction

Intrapartum-related events (birth asphyxia) are the second leading cause of neonatal mortality and are estimated to cause approximately 700 000 deaths annually.^1 2^ Neonatal mortality has declined at a slower pace in sub-Saharan Africa than in the rest of the world. In Uganda the neonatal mortality rate fell from 39 deaths per 1000 live births in 1990 to 20 in 2019.^3^ The country is currently not on track to meet Sustainable Development Goal 3.2, stating that each country should aim for a neonatal mortality rate below 12 by 2030.^4^ Helping Babies Breathe (HBB)^5^ is a basic neonatal resuscitation curriculum for low-resource settings aiming to improve skilled attendance at birth. National roll-out of HBB has been one of the cornerstones to further reduce neonatal mortality.^6 7^

Although the neonate’s transition from oxygenation from the placenta to pulmonary-based oxygenation is an intricate orchestration involving many aspects, for most neonates the process is uncomplicated.^8^ Of all neonates born in the world annually, around 5%–6% (7–9 million) will need neonatal resuscitation at birth.^9^ High-quality resuscitation has the potential to prevent neonatal deaths and complications from birth asphyxia. Positive pressure ventilation (PPV) to ensure inflation of the lungs and onset of alveolar aeration is key to successful resuscitation.^10^ According to international guidelines, PPV should be initiated within 1 min from birth and heart rate assessed after 30–60 s.^11 12^

Video recordings have been suggested to be a valuable tool to evaluate resuscitation performance during neonatal resuscitations.^13 14^ Difficulties to properly perform PPV have been reported even when highly experienced neonatologists resuscitate neonates.^15 16^ In low-resource settings, clinical practices of neonatal resuscitation have rarely been examined. In this prospective observational study, we aimed to detail the crucial events of neonatal resuscitation performed by HBB-trained birth attendants in a low-resource setting. Our primary objective was to investigate the cumulative proportion of ventilation time during the first minute in neonates in need of PPV. The secondary objectives included examining interruptions in PPV and the cause of interruptions.

## Methods

### Study design

This observational study was performed using video recordings of neonatal resuscitations at the Mulago National Referral Hospital from 10 November 2015 to 26 January 2016. The sample was limited to the neonatal resuscitation attempts recorded during the study period for practical reasons, and given similar studies a sample size of 140 resuscitation attempts were considered acceptable. Inclusion criteria were neonates needing PPV support taken to the resuscitation table where birth attendants, HBB-trained or certified by similar neonatal resuscitation programmes, initiated the resuscitation. Exclusion criteria were recordings of resuscitations with bad video quality, recordings where no resuscitation was done, recordings where there were no apparent signs of life after resuscitation, resuscitations using laryngeal mask airway and resuscitations of neonates weighing ≤1000 g. We also excluded resuscitations where advanced resuscitation practices were initiated immediately on arrival to the table. Cases missing a report form or a video recording were also excluded.

### Study setting

The Mulago National Referral Hospital is located in the capital city Kampala. The study was conducted at the high-risk labour ward and operating theatre, Department of Obstetrics and Gynaecology. The labour ward had 16 beds with one resuscitation table, with approximately 40–60 vaginal or assisted deliveries daily. On average, 15–20 caesarean sections were carried out per day in the two theatres, each with a neonatal resuscitation table. All three resuscitation tables were equipped with basic resuscitation equipment, including 250 mL self-inflating bags and masks of sizes 0 and 1 for PPV, as well as nasal cannulas with oxygen and bulb syringes for suction. Face mask was the most common tool used for PPV, while laryngeal mask airway was sometimes used.

### Patient and public involvement

It was deemed not appropriate to involve patients or the public in the design, or conduct, or reporting or dissemination plans for the study.

### Study procedures and data collection

All neonates needing PPV placed on the open resuscitation table during the study period were video-recorded and assessed for eligibility. The research team estimated the time from birth to arrival to the resuscitation table to a mean time of 90 s in the labour ward and 30 s in theatre after observations and discussions with local birth attendants and hospital officials. The cameras (D-Link DCS-820L) were equipped with motion detection and started recording when a neonate was brought to the resuscitation table. Only the neonate and hands of the birth attendants were visible. The data from the cameras were downloaded and stored on securely kept external hard drives.

The free-of-charge iPad application NeoTapAS (Advanced Support)^17^ was used to log and time the various events observed on the video recordings. There are two versions: one for smartphones (NeoTapLS) and one for tablets (NeoTapAS). Studies have shown NeoTap to be a reliable, accurate and fast aid in assessing neonatal resuscitations.^18–21^ Two investigators (DH and DM) reviewed and logged the times and events for each included resuscitation. This included the duration of time of PPV, stimulation and suction. An interruption in ventilation was any type of activity that was not PPV after the PPV had started, for example stimulation or suction. A suction event was defined as when the bulb syringe was inserted in a nostril or the mouth and then removed. The head and mask positions during PPV were observed, and if not the recommended neutral or sniffing position or accurate mask placement described in the HBB were present this was noted together with if any type of heart assessment was made. If discrepancies arouse between the two logs, the first investigator (DH) reviewed the resuscitation again and made a final decision. This occurred for 25 resuscitations. From the NeoTapAS application the data were transferred to R^22^ for statistical analysis.

Written and informed consent was obtained from birth attendants working at the labour ward and theatre during the study period. Qualifications, neonatal resuscitation training and whether they used the HBB action plan during their daily practice were noted. Deferred consent was obtained from the mothers of neonates being resuscitated before storing or viewing the corresponding video recording. A case report form containing the neonate’s time of birth, mode of delivery, who delivered the baby, birth weight, gender, birth type (singleton or twin), Apgar score at 1 and 5 min, any abnormal features, and the mother’s sociodemographics, antenatal and natal history was filled in after the resuscitation if deferred consent was obtained.

### Statistical analysis

Descriptive information regarding study selection process is presented along with descriptive statistics of mothers, neonates and the birth attendants in number (%). Due to the different locations and modes of birth, resuscitations taking place in the labour ward were compared with resuscitations taking place in theatre. The primary and secondary outcomes were analysed through descriptive statistics with number (%) for categorical variables and median (IQR) and compared through Fisher’s exact test and Wilcoxon rank-sum test due to the dependent variable having low number of counts and being non-parametric. All quantitative data were analysed in R.^22^ P<0.05 was considered statistically significant.

## Results

**Figure 1:**
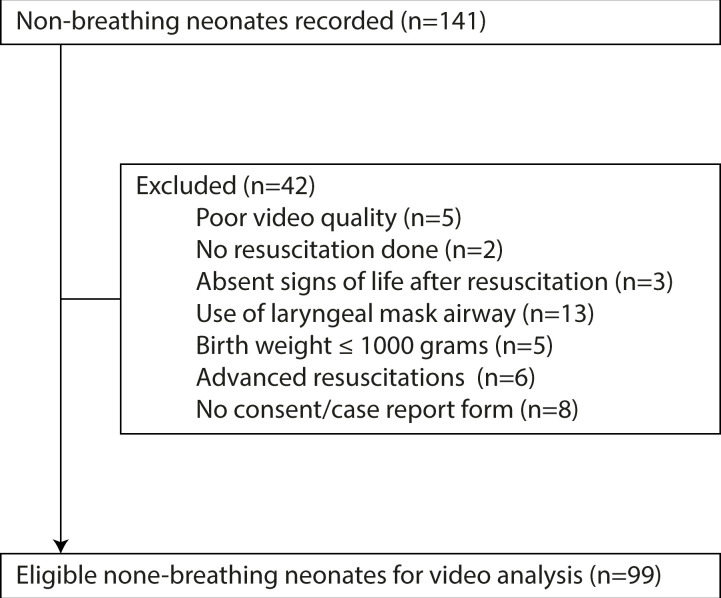
Study profile.

In total 141 resuscitation attempts were recorded, 42 of these were excluded primarily due to use of laryngeal mask airway and no consent or case report form, which resulted in 99 video recordings eligible for timing and logging (figure 1).

### Baseline characteristics

Pregnancy complications were common (table 1). The vaginal and caesarean delivery mode ratio was 1:1. The median number of neonates with Apgar score 0–3 at 1 and 5 min was 28 (28%) and 6 (6%), respectively. A shift in overall median Apgar score between the first and fifth minute from 4–6 to ≥7 was observed. Most neonates weighed ≥3000 g. All birth attendants had at least a midwifery certificate, and all but four birth attendants claimed to use the HBB action plan during resuscitations.

**Table 1:** Characteristics of included mothers, neonates and birth attendants

Mothers	n=99 n (%)	Neonates	n=99 n (%)	Birth attendants	n=45 n (%)
Age (years)		Gender		Age (years)	
15–19	15 (15)	Female	52 (52)	20–30	14 (31)
20–24	42 (42)	Male	47 (47)	31–41	21 (47)
30–34	10 (10)	Birth type		42–52	10 (23)
35–49	11 (11)	Singleton	90 (90)	Qualification level	
Antenatal attendance		Twins	9 (9)	Degree nurse	2 (4)
Yes	96 (97)	Apgar score at 1 min		Diploma midwife	27 (60)
No	3 (3)	0–3	28 (28)	Certificate midwife	16 (36)
Birth order		4–6	58 (58)	Attended HBB refresher course	
First child	38 (38)	≥7	12 (12)	Yes	27 (60)
Second child	19 (19)	Apgar score at 5 min†		No	18 (40)
Third child or later	42 (42)	0–3	6 (6)	Time since last HBB refresher course	
Pregnancy complications		4–6	45 (45)	≤6 months	10 (37)
Obstructed labour	14 (14)	≥7	48 (48)	6–12 months	4 (15)
Fetal distress	4 (4)	Birth weight in grams		≥12 months	13 (48)
Pre-eclampsia/eclampsia	3 (3)	1000–1499	7 (7)	Reasons for not attending HBB refresher course	
Breech	5 (5)	1500–1999	9 (9)	I have adequate knowledge	8 (18)
Cord prolapse	2 (2)	2000–2999	26 (26)	No time	9 (20)
Oligohydramnios	6 (6)	≥3000	56 (56)	No finances	10 (22)
Preterm birth	1 (1)			No such course was offered	4 (9)
Other*	16 (16)			No opportunity	5 (11)
No complications	48 (48)			No answer	2 (4)
Mode of delivery				Used the HBB action plan during resuscitations	
Spontaneous vaginal delivery	48 (48)			Yes	41 (91)
Caesarean section	48 (48)			No	4 (9)
Instrumental delivery	3 (3)				

*Other: cervical dystocia, bleeding conditions such as placenta previa or placental abruption, and delayed second stage of labour, which in 14 out of 16 occurrences resulted in a caesarean section.

†Apgar score at 10 min was not included as most attention is given to the scoring at 5 min.

HBB, Helping Babies Breathe.

### Ventilation

**Figure 2:**
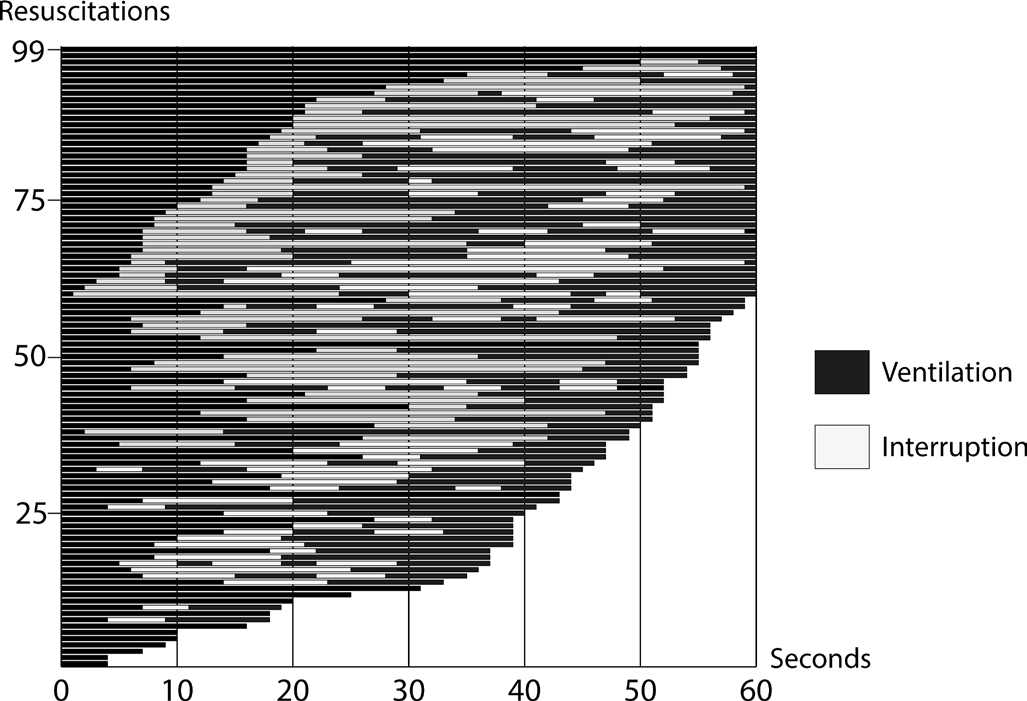
Duration of ventilation and interruptions in ventilation (in seconds) during the first minute after initiating positive pressure ventilation in 99 resuscitations.

**Figure 3:**
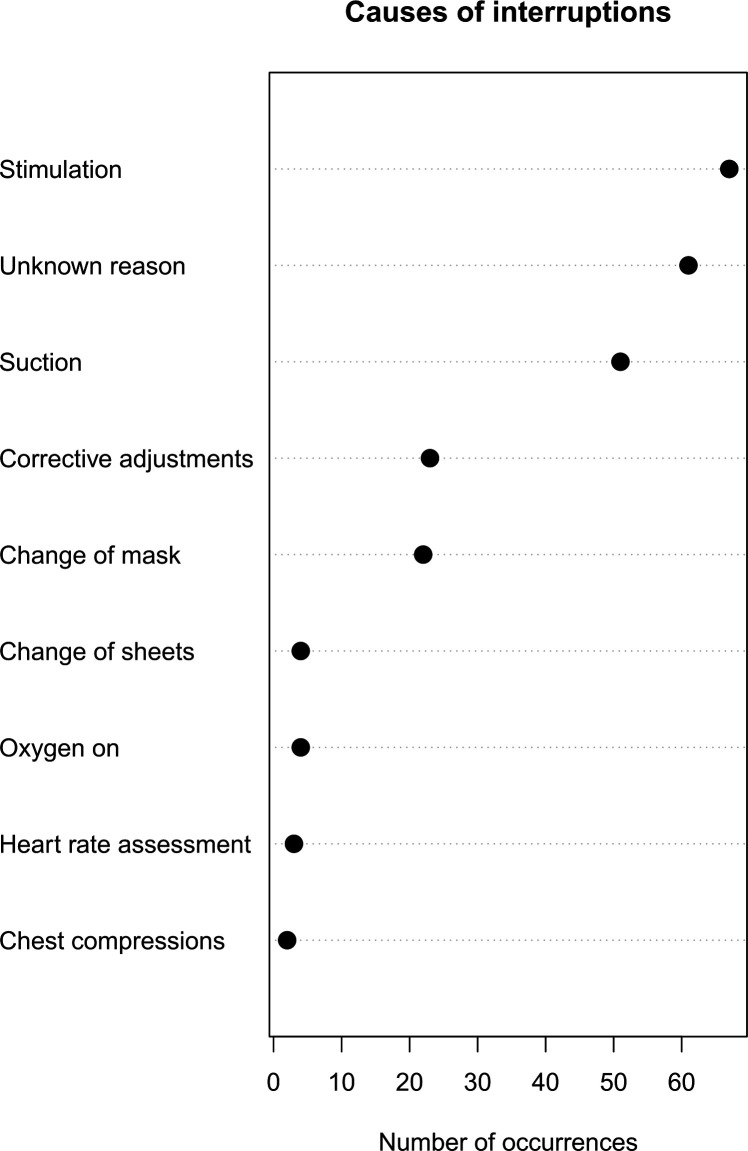
Cause of interruptions during the first 2 min of initiated positive pressure ventilation.

The median (IQR) time from birth to initiation of PPV was 137 (94–168) s and from arrival to the table was 66 (44–102) s (table 2). There was minimal PPV during the first minute after arrival to the resuscitation table. In resuscitations taking place in the labour ward, there were 0 (0–10) s of PPV and 0 (0–12) s in the theatre, and at 2 min resuscitations taking place in the labour ward had included 19 (7–32) s of PPV while resuscitations in the theatre had 18 (8–25) s of ventilation. Overall, during the first 2 min after arriving to the table, there had only been 21 (12–36) s of PPV, with no significant difference between the place of birth. Additionally, approximately a third of the neonates did not have the recommended neutral to sniffing head position during ventilation and the resuscitation was mainly performed by a single birth attendant. The duration of interruptions in ventilations at 1 min after initiating PPV for all resuscitations was 25 (15–35) s and 29 (24–38) s of interruption, respectively (figure 2 and table 2). Interruptions persisted, and for the first 2 min after initiating PPV there were 72 (47–89) s of interruption for both groups. Stimulation (28%, n=67), no apparent reason (26%, n=61) and suction (22%, n=51) were the primary causes of interruptions, while corrective adjustments of the position of the neonate and change of face mask were also common causes (figure 3).

**Table 2:** Resuscitation procedures during the first and second minute after arriving at the resuscitation table

Place of birth	Total (n=99)	Labour ward (n=51)	Theatre (n=48)	P value
Time	Seconds (IQR)	Seconds (IQR)	Seconds (IQR)	
Ventilation				
Time to initiation of PPV from birth*	137 (94–168)	161 (142–197)	92 (68–113)	<0.001
Time to initiation of PPV after arrival to the table	66 (44–102)	71 (52–107)	62 (38–83)	0.126
Total duration of PPV at 1 min	0 (0–10)	0 (0–10)	0 (0–12)	0.159
Total duration of PPV at 2 min	18 (7–30)	19 (7–32)	18 (8–25)	0.623
Total duration of PPV at first 2 min	21 (12–36)	19 (9–43)	23 (14–32)	0.616
Total duration of interruption at 1 min of ventilation	28 (18–37)	25 (15–35)	29 (24–38)	0.132
Total duration of interruption at first 2 min of ventilation	72 (47–89)	68 (45–84)	81 (57–93)	0.059
Stimulation				
Total stimulation time at 1 min	9 (3–18)	5 (0–9)	16 (11–21)	<0.001
Total stimulation time at 2 min	5 (0–10)	4 (0–8)	2 (6–10)	0.125
Total stimulation time at first 2 min	16 (7–26)	8 (4–21)	24 (15–29)	<0.001
Suction				
Total suction time at 1 min	21 (8–29)	23 (10–33)	18 (6–25)	0.136
Total suction time at 2 min	7 (0–20)	4 (0–21)	9 (0–19)	0.244
Total suction time at first 2 min	28 (15–43)	28 (16–44)	29 (14–43)	0.737
**Frequency**	**n (%)**	**n (%)**	**n (%)**	
Only one birth attendant	62 (63)	29 (57)	33 (69)	0.299
Ventilation procedure				
Not recommended head position	31 (31)	8 (16)	23 (48)	0.001
Not accurate mask position	16 (16)	5 (10)	11 (23)	0.103
Heart rate assessment				
By auscultation	1 (1)	1 (2)	0 (0)	1
By umbilical cord palpation	5 (5)	1 (2)	4 (8)	0.196
By chest palpation	11 (11)	9 (18)	2 (4)	0.052

Results are presented as median seconds (IQR) or n (%) and compared with Wilcoxon rank-sum test for continuous and Fisher’s exact test for categorical variables.

*For resuscitations taking place at the labour ward (n=51) and theatre (n=48), the estimated time from birth to resuscitation was 90 s and 30 s, respectively. All other measurements in the table are from when the neonate arrives at the resuscitation table.

PPV, positive pressure ventilation.

### Stimulation and suction

Stimulation time at 1 min was significantly lower for the resuscitations taking place in the labour ward 5 (0–9) s vs 16 (11–21) s (p<0.001); stimulation was less present at 2 min (table 2). Overall, for the first 2 min after arriving to the table, the duration of stimulation was 16 (7–26) s. Suction was prevalent for the whole resuscitation but particularly at 1 min; the cumulative suction time for both groups was 21 (8–29) s, with a median of 5 (2–8) suction events with bulb syringes per neonate. At 2 min there was less duration of suction in both groups. A median of 8 (4–13) separate suction events per neonate which led to a cumulative time effort of 28 (15–43) s was recorded during the first 2 min after arriving to the resuscitation table. Lastly, only 17% of the resuscitations included any form of heart rate assessment.

## Discussion

This observational study used video recordings of neonatal resuscitations in a country with high neonatal mortality. Our findings demonstrate a low adherence to standard resuscitation practices in 2015–2016, with inadequate initiation and duration of PPV, tendency to overstimulate and excessive focus on suction regardless of mode and place of birth. This adds to the comparable resuscitation patterns with insufficient or incorrect ventilation efforts found in a similar setting in Mozambique,^23^ while a study in Nepal concluded that none of the examined resuscitations met the golden minute guideline standard.^24^ However, failing to initiate proper ventilation within the first minute and applying suboptimal PPV are also common in high-resource settings.^25–27^ Specifically, a study from a tertiary hospital in Norway reports a median time from arriving to the table to initiation of PPV of 42 s and 56% of neonates received PPV, with a 60% ventilation fraction during the first 30 s.^25^ The time to initiation of PPV was considerably longer in our study and does not commence within the recommended golden minute after birth. However, primarily due to logistical reasons, the time from birth to resuscitation was significantly longer for neonates in the labour ward. In general it seems that ventilation during resuscitations tends not to be commenced within the recommended time in both high-resource and low-resource settings.

In our study, the high number and relatively long duration of interruptions were reflected in the low ventilation time during the first minute of ventilation not meeting the recommended 60 s of continuous PPV before assessing the adequacy of ventilation, with no significant difference between resuscitations taking place in the labour ward and theatre. We build on the previous literature by showcasing what the actual causes of those interruptions were. In our study, most interruptions were due to stimulation, unknown reasons and suctioning, which should not disrupt PPV. In the Norwegian study^25^ the main reasons for ventilation interruptions were for adjustments for optimising ventilation, heart rate evaluation and stimulation. This was often not the case in our study. It is, however, important to acknowledge and stress the difference in staff quantity and resources between these two settings. According to a qualitative study from Tanzania the main reasons, as per the birth attendants themselves, for delay or interruptions of PPV were fear of doing a poor job in an acute situation and difficulties in assessing the neonate and in taking appropriate action.^28^ Similar findings have been reported from high-resource settings as well.^29^ It is highly likely that the lack of adherence to guidelines in our study stems from both a lack of knowledge and professional confidence, compounded by the limited resources and low number of staff.

Our study reveals excessive amounts of both stimulation and suctioning, the former being the main reason for ventilation interruptions, adding strength to similar findings in other settings.^25 30 31^ In particular, neonates in need of resuscitation in the theatre were stimulated for a longer duration than those in the labour ward; in part the discrepancy could be caused by a difference in perception of the need for stimulation after birth depending on the mode of delivery. Intriguingly, Wrammert *et al*^32^ showed excessive usage of both stimulation and suctioning as the main reason to ventilation delay and halt in resuscitations conducted in Nepal, and proposed that the continuation of the phenomenon was due to difficulties of abandoning a tradition of suctioning of non-breathing neonates before the implementation of HBB or similar educational programmes. Further, it is understandable that in an acute situation where time is of the essence, one might want to use other strategies if ventilation seems fruitless. Excessive usage of stimulation and suctioning in our study could be explained by this need, requiring special attention when developing educational programmes. Lastly, heart rate assessments were rarely done, a vital component of neonatal resuscitation often underused in low-resource settings.^33^

Almost all birth attendants claimed to be using the HBB action plan routinely when working with neonatal resuscitation. The results of our study however show a real-world situation where set guidelines were not followed. The need for and the beneficial effects of acquired skills and knowledge to be frequently updated, practised and evaluated are clear, while similarly important is to increase staff numbers and forming close working professional teams for resuscitations.^24 34–36^

### Strengths and weaknesses

Most limitations of this study are inherent to post-hoc video analysis. First, the factual time spent between birth and arrival to the table was estimated and may not reflect the correct time in each case. Second, assessing recorded resuscitation procedures relies on the clinical expertise of the reviewer, and distinguishing between different procedures is not always clear. Third, although an improvement in Apgar scores from 1 to 5 min was observed, it was not possible to evaluate the effectiveness of PPV since no certain observation of tidal volumes or pulse oximetry was performed. However, by using video recordings the most critical aspects of actual resuscitation practices can be examined and timely logged and an overall picture formed.

## Conclusion

This study demonstrates real-life neonatal resuscitation practices in 2015–2016 that are not in line with the guidelines, with inadequate initiation and duration of PPV, tendency for overstimulation and excessive focus on suction. There is a strong need for continuous educational efforts and investments in adequate resources to increase the quality of clinical neonatal resuscitation practices in low-resource settings.

## Supplementary Material

Author's manuscript

## Data Availability

All data relevant to the study are included in the article or uploaded as supplementary information. All data relevant to this study are included in the article.
