# Supplementary material for: Neonatal resuscitation practices in Uganda: a video observational study
Source: BMJ Paediatr Open. 2021 Sep 14;5(1):e001092. doi: 10.1136/bmjpo-2021-001092 (PMC8442103; doi:10.1136/bmjpo-2021-001092)
Supplement: Author's manuscript [file bmjpo-2021-001092.draft_revisions.pdf]

BMJ Paediatrics Open

BMJ Paediatrics Open is committed to open peer review. As part of this commitment we make the peer review history of every article we publish publicly available.

When an article is published we post the peer reviewers' comments and the authors' responses online. We also post the versions of the paper that were used during peer review. These are the versions that the peer review comments apply to.

The versions of the paper that follow are the versions that were submitted during the peer review process. They are not the versions of record or the final published versions. They should not be cited or distributed as the published version of this manuscript.

BMJ Paediatrics Open is an open access journal and the full, final, typeset and author-corrected version of record of the manuscript is available on our site with no access controls, subscription charges or pay-per-view fees (http://bmjpaedsopen.bmj.com).

If you have any questions on BMJ Paediatrics Open's open peer review process please email info.bmjpo@bmj.com

BMJ Paediatrics Open

Examining neonatal resuscitation practices in Uganda - a video-observational study

Journal:	*BMJ Paediatrics Open*
Manuscript ID	bmjpo-2021-001092
Article Type:	Original research
Date Submitted by the Author:	13-Mar-2021
Complete List of Authors:	Helldén, Daniel ; Karolinska Institute, Global Public Health<br>Myrnerts Höök, Susanna; Karolinska Institute, Global Public Health; University of Bergen Faculty of Medicine and Dentistry, Center for International Health<br>Pejovic, Nicolas; Karolinska Institute, Global Public Health; Sachs' Children and Youth Hospital<br>Mclellan, Dan; Karolinska Institute, Global Public Health<br>Lubulwa, Clare; Mulago Specialized Women and Neonatal Hospital<br>Tylleskär, Thorkild; University of Bergen Faculty of Medicine and Dentistry, Centre for International Health; University of Bergen, Centre for Intervention Science in Maternal and Child Health, Department of Global Public Health and Primary Care<br>Alfven, Tobias; Department of Global Public Health; Sachs' Children and Youth Hospital,
Keywords:	Neonatology, Resuscitation, Health services research

SCHOLARONE™
Manuscripts

I, the Submitting Author has the right to grant and does grant on behalf of all authors of the Work (as defined in the below author licence), an exclusive licence and/or a non-exclusive licence for contributions from authors who are: i) UK Crown employees; ii) where BMJ has agreed a CC-BY licence shall apply, and/or iii) in accordance with the terms applicable for US Federal Government officers or employees acting as part of their official duties; on a worldwide, perpetual, irrevocable, royalty-free basis to BMJ Publishing Group Ltd ("BMJ") its licensees and where the relevant Journal is co-owned by BMJ to the co-owners of the Journal, to publish the Work in this journal and any other BMJ products and to exploit all rights, as set out in our licence.

The Submitting Author accepts and understands that any supply made under these terms is made by BMJ to the Submitting Author unless you are acting as an employee on behalf of your employer or a postgraduate student of an affiliated institution which is paying any applicable article publishing charge ("APC") for Open Access articles. Where the Submitting Author wishes to make the Work available on an Open Access basis (and intends to pay the relevant APC), the terms of reuse of such Open Access shall be governed by a Creative Commons licence – details of these licences and which Creative Commons licence will apply to this Work are set out in our licence referred to above.

Other than as permitted in any relevant BMJ Author's Self Archiving Policies, I confirm this Work has not been accepted for publication elsewhere, is not being considered for publication elsewhere and does not duplicate material already published. I confirm all authors consent to publication of this Work and authorise the granting of this licence.

Confidential: For Review Only

Title: Examining neonatal resuscitation practices in Uganda - a video-observational study

Authors: Daniel Helldén[1]*, Susanna Myrnerts Höök[*1,2,3], Nicolas Pejovic[1,2], Dan McLellan[1], Clare Lubulwa[4], Thorkild Tylleskär[3,5], Tobias Alfvén[1,2]

Affiliations:

[1]Department of Global Public Health, Karolinska Institutet, Stockholm, Sweden

[2]Sachs' Children and Youth Hospital, Stockholm, Sweden

[3]Center for International Health, University of Bergen, Bergen, Norway

[4]Mulago Specialized Women and Neonatal Hospital, Kampala, Uganda

[5]Center for Intervention Science in Maternal and Child Health, University of Bergen, Bergen, Norway

* These authors contributed equally

Keywords: Neonatal resuscitation, positive pressure ventilation, Helping Babies Breathe

Word count

Text: 2449

[1] Affiliation: Department of Global Public Health, Karolinska Institutet, Stockholm, Sweden
Address: Tomtebodavägen 18 A, SE-171 77 Stockholm, Sweden
Email: daniel.hellden@ki.se
Telephone: +46732406555

Confidential: For Review Only

ABSTRACT

Background: Neonatal mortality, often due to birth asphyxia, remains stubbornly high in sub-Saharan Africa. Guidelines for neonatal resuscitation, where achieving adequate positive-pressure ventilation (PPV) is key, has been implemented in low-resource settings. However, the actual clinical practices of neonatal resuscitation have rarely been examined in these settings. The primary aim of this prospective observational study was to detail the cumulative proportion of time with ventilation during the first minute on the resuscitation table of neonates needing PPV at the Mulago National Referral Hospital in Kampala, Uganda.

Methods: From November 2015 to January 2016, resuscitations of non-breathing neonates by birth attendants were video-recorded using motion-sensor cameras. The resuscitation practices were analysed using the application NeoTapAS and the statistical software RStudio.

Results: From 144 recorded resuscitations, 99 were included for analysis. The median (IQR) ventilation fraction during the first minute of ventilation was 0.53 (0.39-0.71). Resuscitations having a ventilation fraction ≥50 percent (n=58) were compared to resuscitations with a ventilation fraction <50 percent (n=41). The time to initiation of PPV were 66 (42-102) seconds overall, and after initiating PPV the duration of interruptions during the first minute were 20 (12-26) seconds and 39 (34-43) seconds for respective group (P<0.001). The majority of interruptions were caused by stimulation (28%), unknown reason (25%) and suction (22%).

Conclusions: Our findings show a low adherence to standard resuscitation practices in 2015-2016. This emphasizes the need for continuous educational efforts and investments in staff and adequate resources to increase the quality of clinical neonatal resuscitation practices in low-resource settings.

What is known about the subject?

- Timely initiation and duration of adequate ventilation is key to high-quality neonatal resuscitation

- Educational programs such as Helping Babies Breathe have been implemented to improve clinical practice in neonatal resuscitation

What this study adds?

- Analysis of video-recordings of actual neonatal resuscitation practices shows that they suffer from inadequate initiation, duration, and quality of positive-pressure ventilation, tendency for overstimulation, and excessive focus on suction.

- There is a strong need for continuous educational efforts and investments in staff and resources to increase quality of neonatal resuscitation practices in low-resource settings.

https://mc.manuscriptcentral.com/bmjpo

Confidential: For Review Only

INTRODUCTION

Intrapartum-related events (birth asphyxia) is the second leading cause of neonatal mortality and is estimated to cause approximately 700 000 deaths annually [1,2]. Neonatal mortality has declined at a slower pace in sub-Saharan Africa than in the rest of the world. In Uganda the neonatal mortality rate fell from 39 in 1990 to 20 in 2019 [3]. The country is currently not on track to meet the Sustainable Development Goal 3.2 that states that each country should aim for a neonatal mortality rate below 12 by 2030 [4]. Helping Babies Breathe (HBB) [5] is a basic neonatal resuscitation curriculum for low-resource settings aiming to improve skilled attendance at birth. National roll-out of HBB has been one of the cornerstones to further the decline of neonatal mortality [6,7].

Although the neonate´s transition from oxygenation from the placenta to pulmonary-based oxygenation is an intricate orchestration involving many aspects, for most neonates the process is uncomplicated [8]. Of all neonates born in the world annually, around 5-6 percent (7-9 million) will need neonatal-resuscitation at birth [9]. High quality resuscitation has the potential to prevent neonatal deaths and complications from birth asphyxia. Positive-pressure ventilation (PPV) to ensure inflation of lungs and onset of alveolar aeration is key to successful resuscitation [10]. According to international guidelines, PPV should be initiated within one minute from birth and heart rate assessed after 30-60 seconds [11,12].

Video recordings have been suggested to be a valuable tool to evaluate resuscitation performance during neonatal resuscitations [13,14]. Difficulties to properly perform PPV have been reported even when highly experienced neonatologists resuscitate neonates [15,16]. In low-resource settings, clinical practices of neonatal resuscitation have rarely been examined. In this prospective observational study, we aimed to detail the crucial events of neonatal resuscitation performed by HBB-trained birth attendants in a low-resource setting. Our primary objective was to investigate the cumulative proportion of ventilation time during the first minute in neonates in need of PPV. Secondary objectives included examining interruptions in PPV and the cause for the interruptions.

METHODS

Study design

This observational study was performed utilizing video recordings of neonatal resuscitations at Mulago National Referral Hospital from 10th November 2015 to 26th January 2016. The sample was limited to the neonatal resuscitation attempts recorded during the study period for practical reasons, and given similar studies, a sample size of 140 resuscitation attempts were considered acceptable. Inclusion criteria were neonates needing PPV support taken to the resuscitation table where birth attendants, HBB trained or certified by similar neonatal resuscitation programmes, initiated the resuscitation. Exclusion criteria were resuscitations using laryngeal mask airway, poor video quality, neonates born weighing ≤1000 grams and resuscitations with no use of mask ventilation. Cases missing case report form or video

Confidential: For Review Only

recording were also excluded.

Study setting

The Mulago National Referral Hospital is located in the capital city Kampala. The study was conducted at the High-risk Labour Ward and Operating Theatre, Department of Obstetrics and Gynaecology. The labour ward had 16 beds with one resuscitation area, and approximately 40-60 vaginal or assisted deliveries daily. On average, 15-20 caesarean sections were carried out per day in the theatre, which was equipped with two operating beds each which a neonatal resuscitation area. All three resuscitation areas were equipped with basic resuscitation equipment including bulb syringes for suction, 250 ml self-inflating bag and masks of size 0 and 1 for PPV as well as nasal cannulas with oxygen.

Patient and public involvement

It was deemed not appropriate to involve patients or the public in the design, or conduct, or reporting or dissemination plans for the study.

Study procedures and data collection

All neonates needing PPV placed on the open resuscitation table during the study period were video recorded and assessed for eligibility. The research team estimated the time from birth to arrival to the resuscitation table to a mean time of 90 seconds in the labour ward and 30 seconds in the theatre after observations and discussions with local birth attendants and hospital officials. The cameras (D-Link DCS 820L) were equipped with motion detection and started recording when a neonate was brought to the resuscitation table. Only the neonate and hands of the birth attendants were visible. The data from the cameras were downloaded and stored on securely kept external hard drives.

The free-of-charge iPad application NeoTapAS (Advanced Support) [17] was used to log and time the various events observed in the video recordings. There are two versions one for smartphones (NeoTapLS) and one for tablets (NeoTapAS). Studies have shown NeoTap to be a reliable, accurate and fast aid in assessing neonatal resuscitations [18–21]. Two investigators (DH and DM) reviewed and logged times and events for each included resuscitation. If discrepancies arouse between the two logs, the first investigator (DH) reviewed the resuscitation again and made a final decision. This occurred for 25 resuscitation attempts. From the NeoTapAS application the data were transferred into R Studio [22] for statistical analysis.

Written and informed consent was obtained from birth attendant working at the labour ward and theatre during the study period. Level of qualification, neonatal resuscitation training and whether they used the HBB action plan during their daily practice were noted. Deferred consent was obtained from the mothers of neonates being resuscitated before storing or viewing the corresponding video recording. A case report form containing the neonate’s time

Confidential: For Review Only

of birth, mode of delivery, who delivered the baby, birth weight, gender, birth type (singleton or twin), Apgar score at 1 and 5 minutes, any abnormal features, the mother's socio-demographics, antenatal and natal history was filled in after the resuscitation if deferred consent was obtained.

Statistical analysis

Descriptive information regarding study selection process is presented along with descriptive statistics of mothers, neonates and the birth attendants in numbers (%) for categorial variables and median (interquartile range) for continuous variables.

Comparison groups were created using the ventilation fraction during the first minute of ventilation. The cumulative time of ventilation efforts during the first 60 seconds after PPV was initiated were transformed into a ventilation fraction for each resuscitation. Resuscitations having a ventilation fraction ≥50 percent were compared to resuscitations with a ventilation fraction <50 percent.

The primary and secondary outcomes were analysed through descriptive statistics of the ventilation fraction during the first minute of initiated PPV and compared through Fishers Exact Test for categorical variables and Wilcox Rank Sum Test for continuous variables, between ventilation fraction ≥50 percent and ventilation fraction <50 percent. All quantitative data were analysed in RStudio [22]. A p-value <0.05 was considered to be statistically significant.

RESULTS

In total 141 resuscitation attempts were recorded, of these 42 were excluded primarily due to use of laryngeal mask airway and no consent or case report form, which resulted in 99 video recordings eligible for timing and logging (**Figure 1**).

Baseline characteristics

Pregnancy complications were common (**Table 1**). Vaginal and caesarean delivery mode ratio was one to one. The median number of neonates with Apgar score 0-3 at minute 1 and minute 5 was 28 (28%) and 6 (6%), respectively. A shift in overall median Apgar score between first and fifth minute from 4-6 to ≥7 was observed. Most neonates weighed ≥3000 grams. All birth attendants had at least a midwifery certificate and all but 4 birth attendants claimed to use the HBB action plan during resuscitations.

Confidential: For Review Only

Table 1. Characteristics of included mothers, neonates and birth attendants.

**Mothers**	Number N = 99 n (%)	**Neonates**	Number N = 99 n (%)	**Birth attendants**	Number N = 45 n (%)
**Age (years)**		**Gender**		**Age (years)**	
15 – 19	15 (15)	Female	52 (52)	20 – 30	14 (31)
20 - 24	42 (42)	Male	47 (47)	31 – 41	21 (47)
30 – 34	10 (10)	**Birth type**		42 – 52	10 (23)
35 – 49	11 (11)	Singleton	90 (90)	**Qualification level**	
**Antenatal attendance**		Twins	9 (9)	Degree nurse	2 (4)
Yes	96 (97)	**Apgar score at 1 minute**		Diploma midwife	27 (60)
No	3 (3)	0 – 3	28 (28)	Certificate midwife	16 (36)
**Birth order**		4 – 6	58 (58)	**Attended HBB refresher course**	
First child	38 (38)	≥ 7	12 (12)	Yes	27 (60)
Second child	19 (19)	**Apgar score at 5 minutes[2]**		No	18 (40)
≥ Third child	42 (42)	0 – 3	6 (6)	**Time since last HBB refresher course**	
**Pregnancy complications**		4 – 6	45 (45)	≤ 6 months	10 (37)
Obstructed labour	14 (14)	≥ 7	48 (48)	6 – 12 months	4 (15)
Fetal distress	4 (4)	**Birth weight in grams**		≥ 12 months	13 (48)
Pre-eclampsia/eclampsia	3 (3)	1000 – 1499	7 (7)	**Reasons for not attending HBB refresher course[3]**	
Breech	5 (5)	1500 – 1999	9 (9)	I have adequate knowledge	8 (18)
Cord prolapse	2 (2)	2000 – 2999	26 (26)	No time	9 (20)
Oligohydramnios	6 (6)	≥ 3000	56 (56)	No finances	10 (22)
Other[1]	17 (17)			No such course was offered	4 (9)
No complications	48 (48)			No opportunity	5 (11)
**Mode of delivery**				**Used the HBB action plan during resuscitations**	
Spontaneous vaginal delivery	48 (48)			Yes	41 (91)
Caesarean section	48 (48)			No	4 (9)
Instrumental delivery	3 (3)				

[1]Other cervical dystocia, bleeding conditions such as placenta previa or placenta abruption, delayed second stage of labour, preterm birth. [2]Apgar score at 10 minutes was not included as most attention is given to the scoring at 5 minutes. [3]2 midwives did not provide a response.

Ventilation

The median (IQR) ventilation fraction during the first minute after initiating ventilation was 0.53 (0.39- 0.71), and there were 58 resuscitations with a ventilation fraction equal or above 50% while 41 ventilated for a cumulative time below 50%. The time from birth to initiation of PPV was 137 (94-168) seconds, and from arrival to table 66 (44-102) seconds (**Table 2**). There was minimal PPV during minute 1 after arrival to the resuscitation table but a significantly higher degree of ventilation during minute 2 in those that had a ventilation fraction of ≥50% with 26 (13-34) versus 12 (3-19) seconds (p-value <0.001). However, during the first 2 minutes after arriving on the table there had only been 31 (15-44) seconds of PPV in the ≥50% group. Additionally, approximately a third of the neonates did not have the recommended neutral to sniffing head position during ventilation and the resuscitation was

Confidential: For Review Only

mainly performed by a single birth attendant. **Figure 2** complements **Table 2**, illustrating the duration of interruptions in ventilations during minute 1 after initiating PPV for all resuscitations, 20 (12-26) and 39 (34-43) seconds of interruption for respective group. Significant interruptions persisted, and for the first 2 minutes after initiating PPV there had been 237 interruptions leading to 72 (47-89) seconds of interruption for both groups. Stimulation 28% (n=67), no apparent reason 26% (n=61) and suction 22% (n=51) were the primary causes for interruptions, while adjustments of the position of the neonate and change of face mask were also common causes (**Figure 3)**.

Table 2. Resuscitation procedures during first and second minute after arriving at the resuscitation table.

**Ventilation fraction 1st minute from ventilation start**	Total (N= 99)	≥50% (N= 58)	<50% (N=41)	p-value
**Time**	**Seconds (IQR)**	**Seconds (IQR)**	**Seconds (IQR)**	
**Ventilation**				
Time to initiation of PPV from birth[1]	137 (94-168)	138 (103-168)	132 (88-163)	0.584
Time to initiation of PPV after arrival to table	66 (44-102)	64 (42-97)	71 (47-102)	0.688
Total duration of PPV minute 1	0 (0-10)	0 (0-15)	0 (0-8)	0.338
Total duration of PPV minute 2	18 (7-30)	26 (13-34)	12 (3-19)	<0.001
Total duration of PPV first 2 minutes	21 (12-36)	31 (15-44)	15 (10-16)	<0.001
Total duration of interruption minute 1 of ventilation	28 (18-37)	20 (12-26)	39 (34-43)	<0.001
Total duration of interruption first 2 minutes of ventilation	72 (47-89)	53 (38-76)	93 (74-97)	<0.001
**Stimulation**				
Total stimulation time minute 1	9 (3-18)	7 (3-15)	15 (6-21)	0.032
Total stimulation time minute 2	5 (0-10)	5 (0-9)	6 (0-10)	0.727
Total stimulation time first 2 minutes	16 (7-26)	14 (7-23)	22 (8-30)	0.077
**Suction**				
Total suction time minute 1	21 (8-29)	21 (7-31)	21 (8-28)	0.966
Total suction time minute 2	7 (0-20)	5 (0-15)	10 (0-26)	0.035
Total suction time first 2 minutes	28 (15-43)	24.5 (12-40)	32 (19-49)	0.150
**Frequency**	**Number (%)**	**Number (%)**	**Number (%)**	
**Ventilation procedure**				
Not accurate head position	31 (31)	16 (28)	15 (37)	0.384
Not accurate mask position	16 (16)	8 (14)	8 (20)	0.581
Only one birth attendant	62 (63)	32 (55)	30 (73)	0.092
**Heart rate assessment**				
By auscultation	1 (1)	1 (2)	0 (0)	1
By palpation	5 (5)	4 (7)	1 (2)	0.400
By chest palpation	11 (11)	8 (14)	3 (7)	0.354

Results are presented as median seconds (IQR) or numbers (%) and compared with Wilcox Rank Sum Test for continuous and Fishers Exact Test for categorical variables. [1] For resuscitations taking place at the delivery room (n=48) and labour ward (n=51) and the estimated time from birth to resuscitation

Confidential: For Review Only

was 30 seconds and 90 seconds respectively. All other measurements in the table are from when the neonate arrives to the resuscitation table.

Stimulation and suction

Stimulation time during minute 1 was significantly lower in the higher ventilation fraction group, with 7 (3-15) versus 15 (6-21) seconds (p-value = 0.032), stimulation was less present in minute 2 (**Table 2**). Overall, for the first 2 minutes after arriving to the table the duration of stimulation was 16 (7-26) seconds. Suction was prevalent for the whole resuscitation but particularly during minute 1, the cumulative suction time for both groups was 21 (8-29) seconds with 536 suction events with bulb syringes in total. During minute 2 there was less duration of suction overall, but in the high ventilation fraction group the time spent on suction was significantly lower 5 (0-15) seconds versus 10 (0-26) seconds (p-value=0.035). In total, 865 separate suctions events with a cumulative time effort of 28 (15-43) seconds were recorded during the first 2 minutes after arriving to the resuscitation table. Lastly, only 17% of the resuscitations included any form of heart rate assessment.

DISCUSSION

This observational study used video recordings of neonatal resuscitations in a country with high a neonatal mortality. Our findings demonstrate a low adherence to standard resuscitation practices in 2015-2016 with inadequate initiation, duration and quality of PPV, tendency for overstimulation, and excessive focus on suction. Comparable resuscitation patterns with insufficient or incorrect ventilation efforts were found in a similar setting in Mozambique [23], while a study in Nepal concluded that none of the examined resuscitations met the golden minute guideline standard [24]. However, failing to initiate proper ventilation within the first minute and applying suboptimal PPV is also common in high-resource settings [25–27]. Specifically, a study from a tertiary hospital in Norway report a median time from arriving to the table to initiation of PPV of 42 seconds and 56% of neonates received PPV with a 60% ventilation fraction during the first 30 seconds. [25] The time to initiation of PPV was considerably longer in our study, and ventilation fraction during the first minute of ventilation lower. In general, it seems that ventilation during resuscitations tend not to be commenced within the recommended time in both high- and low-resource settings. The introduction of a laryngeal mask airway in neonatal resuscitation performed by midwives holds some promise in low- resource settings to provide safe PPV in case of failure to establish effective PPV with face mask [28].

In our study, the high number and relatively long duration of interruptions led to the low ventilation time during both the first and second minute of ventilation. Most interruptions were due to stimulation, unknown reasons, and suctioning, which should not disrupt PPV. In the Norwegian study [25] the main reasons for ventilation interruptions were for adjustments for optimising ventilation, heart rate evaluation, and stimulation. This was often not the case in our study. It is, however, important to acknowledge and stress the difference in staff

Confidential: For Review Only

quantity and resources between these two settings. According to a qualitative study from Tanzania the main reasons, as per the birth attendants themselves, for delay or interruptions of PPV were fear of doing a poor job in an acute situation, difficulties in assessing the neonate and in taking appropriate action [29]. Similar findings have been reported from high-resource settings as well [30]. It is highly likely that the lack of adherence to guidelines in our study stems from both a lack of knowledge and professional confidence, compounded by the limited resources and low number of staff.

Our study reveals excessive amounts of both stimulation and suctioning, the former being the main reason for ventilation interruptions, similar to other studies [25,31,32]. Intriguingly, Wrammert et al. [33] showed excessive usage of both stimulation and suctioning as main reasons to ventilation delay and halt in resuscitations conducted in Nepal, and proposed that the continuation of the phenomenon was due to difficulties of abandoning a tradition of suctioning of non-breathing neonates before the implementation of HBB or similar educational programs. Further, it is understandable that in an acute situation where time is of the essence, one might want to utilize other strategies if ventilation seems fruitless. Excessive usage of stimulation and suctioning in our study could be explained by this need, requiring special attention when developing educational programs. Lastly, heart rate assessments were rarely done, a vital component of neonatal resuscitation often underused in low-resource settings [34].

Almost all birth attendants claimed to be using the HBB action plan routinely when working with neonatal resuscitation. The results, however, display a situation where set guidelines were not followed. The need for and beneficial effects of acquired skills and knowledge to be frequently updated, practiced and evaluated is clear while similarly important is to increase staff numbers and forming close working professional teams for resuscitations [24,35–37].

Strengths and weaknesses

There are a number of limitations with this study, most of them inherent with post-hoc video analysis in a low-resource setting. First, the factual time spent between birth and arrival of the neonate to the resuscitation area is estimated and may not reflect the true time in each resuscitation procedures. Second, assessing recorded resuscitation procedures relies on the clinical expertise of the reviewer, and distinguishing between different procedures is not always clear. Third, though an improvement in Apgar scores from minute 1 to minute 5 was observed, it was not possible to evaluate the effectiveness of PPV since no certain observation of tidal volumes or pulse oximetry was available. However, by using video recordings to assess the resuscitation the most critical aspects of actual resuscitation practices can be examined and timely logged, and an overall picture formed.

Confidential: For Review Only

Conclusion

This study demonstrates real life neonatal resuscitation practices in 2015-2016 that are not in line with guidelines with inadequate initiation, duration, and quality of PPV, tendency for overstimulation and excessive focus on suction. There is a strong need for continuous educational efforts and investments in staff and adequate resources to increase the quality of clinical neonatal resuscitation practices in low-resource settings.

Acknowledgements

We would like to give thanks to all participants of this study. Our greatest thanks to the staff at the Maternity and Special Care Unit of Mulago National Referral Hospital, Kampala, Uganda, for their amazing struggle to improve the care of neonates.

Contributors

DH was responsible for data analysis, interpretation of data and for the writing process including writing a first draft of the manuscript and approval of the final draft. SMH was responsible for the study design and acquisition of data from video recordings. She revised the manuscript and approved the final draft. NJP contributed to study design and acquisition of data. He revised the manuscript and approved the final draft. DM assisted in data analysis and writing process, revised the manuscript and approved the final draft. CL contributed to study design, was responsible for preparation of the study site and collecting the video recordings. She revised the manuscript and approved the final draft. TT contributed to study design, interpretation of data, manuscript revision and approved the final draft. TA contributed to the study design, data analysis, manuscript revision and approval of the final draft.

Funding

The study was funded by the Norwegian Government through the Norwegian Quota Scholarship scheme and by in-kind contributions from the University of Bergen, Norway, Makerere University, Uganda, and the Karolinska Institutet, Sweden.

Competing interests

The authors have no financial relationships relevant to this article to disclose. SMH, NJP, CL, TT and TA are co-founders of the non-profit organisation Tap4Life (www.tap4life.org), which produced the free-of-charge application NeoTap. The SMH, NJP, CL, TT and TA do not get any salary from Tap4Life.

Patient consent for publication

Not required.

Ethics approval

Confidential: For Review Only

Ethical approval of the study was received from the Mulago Hospital Research and Ethics Committee and the Uganda National Council of Science and Technology (MREC:841). The Norwegian research ethics committee exempted it from evaluation since it was deemed a quality assurance study.

Data availability statement

All data relevant to this study are included in the article.

References

1 Ehret DY, Patterson JK, Bose CL. Improving Neonatal Care: A Global Perspective. Clin. Perinatol. 2017;**44**:567–82. doi:10.1016/j.clp.2017.05.002

2 Liu L, Oza S, Hogan D, *et al.* Global, regional, and national causes of child mortality in 2000-13, with projections to inform post-2015 priorities: an updated systematic analysis. *Lancet* 2015;**385**:430–40. doi:10.1016/S0140-6736(14)61698-6

3 UN Inter-agency Group for Child Mortality Estimation. Levels & Trend in Child Mortality: Report 2020. New York: 2020. https://www.unicef.org/media/79371/file/UN-IGME-child-mortality-report-2020.pdf.pdf

4 Hug L, Alexander M, You D, *et al.* National, regional, and global levels and trends in neonatal mortality between 1990 and 2017, with scenario-based projections to 2030: a systematic analysis. *Lancet Glob Heal* 2019;**7**:e710–20. doi:10.1016/S2214-109X(19)30163-9

5 Kamath-Rayne BD, Thukral A, Visick MK, *et al.* Helping Babies Breathe, second edition: A model for strengthening educational programs to increase global newborn survival. *Glob Heal Sci Pract* 2018;**6**:538–51. doi:10.9745/GHSP-D-18-00147

6 Reisman J, Arlington L, Jensen L, *et al.* Newborn resuscitation training in resource-limited settings: A systematic literature review. Pediatrics. 2016;**138**. doi:10.1542/peds.2015-4490

7 Kamath-Rayne BD, Berkelhamer SK, Kc A, *et al.* Neonatal resuscitation in global health settings: An examination of the past to prepare for the future. *Pediatr Res* 2017;**82**:194–200. doi:10.1038/pr.2017.48

8 Morton SU, Brodsky D. Fetal Physiology and the Transition to Extrauterine Life. Clin. Perinatol. 2016;**43**:395–407. doi:10.1016/j.clp.2016.04.001

9 UNICEF. State of the World’s Children 2019: Children, food and nutrition. New York: 2019. https://www.unicef.org/reports/state-of-worlds-children-2019

10 Hooper SB, Siew ML, Kitchen MJ, *et al.* Establishing functional residual capacity in the non-breathing infant. Semin. Fetal Neonatal Med. 2013;**18**:336–43. doi:10.1016/j.siny.2013.08.011

Confidential: For Review Only

11 Perlman JM, Wyllie J, Kattwinkel J, *et al.* Part 7: Neonatal resuscitation 2015 international consensus on cardiopulmonary resuscitation and emergency cardiovascular care science with treatment recommendations. *Pediatrics* 2015;**136**:S120–66. doi:10.1542/peds.2015-3373D

12 World Health Organization. Guidlines on basic newborn resucitation. Geneva: 2012. https://apps.who.int/iris/bitstream/handle/10665/75157/9789241503693_eng.pdf?sequence=1

13 Carbine DN, Finer NN, Knodel E, *et al.* Video Recording as a Means of Evaluating Neonatal Resuscitation Performance. *Pediatrics* 2000;**106**:654–8. doi:doi: 10.1542/peds.106.4.654

14 Schilleman K, Siew ML, Lopriore E, *et al.* Auditing resuscitation of preterm infants at birth by recording video and physiological parameters. *Resuscitation* 2012;**83**:1135–9. doi:10.1016/j.resuscitation.2012.01.036

15 Schmölzer GM, Dawson JA, Kamlin COF, *et al.* Airway obstruction and gas leak during mask ventilation of preterm infants in the delivery room. *Arch Dis Child Fetal Neonatal Ed* 2011;**96**:F254-7. doi:10.1136/adc.2010.191171

16 Moshiro R, Perlman JM, Kidanto H, *et al.* Predictors of death including quality of positive pressure ventilation during newborn resuscitation and the relationship to outcome at seven days in a rural Tanzanian hospital. *PLoS One* 2018;**13**:e0202641. doi:10.1371/journal.pone.0202641

17 Tap4Life. http://tap4life.org/

18 Myrnerts Höök S, Pejovic NJ, Marrone G, *et al.* Accurate and fast neonatal heart rate assessment with a smartphone-based application – a manikin study. *Acta Paediatr Int J Paediatr* 2018;**107**:1548–54. doi:10.1111/apa.14350

19 Johnson PA, Morina N, O'Reilly M, *et al.* Evaluation of a Tap-Based Smartphone App for Heart Rate Assessment During Asphyxia in a Porcine Model of Neonatal Resuscitation. *Front Pediatr* 2019;**7**:453. doi:10.3389/fped.2019.00453

20 Binotti M, Cavallin F, Ingrassia PL, *et al.* Heart rate assessment using NeoTapAdvancedSupport: A simulation study. *Arch Dis Child Fetal Neonatal Ed* 2019;**104**:F440–2. doi:10.1136/archdischild-2018-315408

21 Myrnerts Höök S, Pejovic NJ, Cavallin F, *et al.* Smartphone app for neonatal heart rate assessment: an observational study. *BMJ Paediatr Open* 2020;**4**:e000688. doi:10.1136/bmjpo-2020-000688

22 RStudio Team. RStudio: Integrated Development for R. 2019.

23 Trevisanuto D, Bertuola F, Lanzoni P, *et al.* Effect of a neonatal resuscitation course on healthcare providers' performances assessed by video recording in a low-resource setting. *PLoS One* 2015;**10**:1–12. doi:10.1371/journal.pone.0144443

24 Kc A, Wrammert J, Clark RB, *et al.* Reducing perinatal mortality in Nepal using helping

Confidential: For Review Only

babies breathe. *Pediatrics* 2016;**137**:e20150117. doi:10.1542/peds.2015-0117

25 Skåre C, Boldingh AM, Nakstad B, *et al.* Ventilation fraction during the first 30 s of neonatal resuscitation. *Resuscitation* 2016;**107**:25–30. doi:10.1016/j.resuscitation.2016.07.231

26 Bjorland PA, Ersdal HL, Øymar K, *et al.* Compliance with guidelines and efficacy of heart rate monitoring during newborn resuscitation: A prospective video study. *Neonatology* 2020;**117**:175–81. doi:10.1159/000506772

27 Niles DE, Cines C, Insley E, *et al.* Incidence and characteristics of positive pressure ventilation delivered to newborns in a US tertiary academic hospital. *Resuscitation* 2017;**115**:102–9. doi:10.1016/j.resuscitation.2017.03.035

28 Pejovic NJ, Myrnerts Höök S, Byamugisha J, *et al.* A Randomized Trial of Laryngeal Mask Airway in Neonatal Resuscitation. *N Engl J Med* 2020;**383**:2138–47. doi:10.1056/nejmoa2005333

29 Moshiro R, Ersdal HL, Mdoe P, *et al.* Factors affecting effective ventilation during newborn resuscitation: a qualitative study among midwives in rural Tanzania. *Glob Health Action* 2018;**11**:1423862. doi:10.1080/16549716.2018.1423862

30 Ljungblad LW, Skovdahl K, McCormack B, *et al.* Balancing life and death during the golden minute – midwives' experiences of performing newborn resuscitation. *J Multidiscip Healthc* 2020;**13**:943–52. doi:10.2147/JMDH.S268959

31 Bansal SC, Nimbalkar AS, Patel D V., *et al.* Current Neonatal Resuscitation Practices among Paediatricians in Gujarat, India. *Int J Pediatr* 2014;**2014**:1–7. doi:10.1155/2014/676374

32 Adams E, O´Neal P, Hanson L, *et al.* Airway clearance of the term newborn. *MCN Am J Matern Child Nurs* 2018;**43**:306–12. doi:10.1097/NMC.0000000000000473

33 Wrammert J, Zetterlund C, Ashish KC, *et al.* Resuscitation practices of low and normal birth weight infants in Nepal: An observational study using video camera recordings. *Glob Health Action* 2017;**10**:1322372. doi:10.1080/16549716.2017.1322372

34 Johnson PA, Schmölzer GM. Heart Rate Assessment during Neonatal Resuscitation. *Healthcare* 2020;**8**:43. doi:10.3390/healthcare8010043

35 Hodgins S. Helping babies breathe - beyond training. *Glob Heal Sci Pr* 2018;**6**:402–4. doi:10.1016/j.jpeds.2012.03.030

36 Bang A, Patel A, Bellad R, *et al.* Helping Babies Breathe (HBB) training: What happens to knowledge and skills over time? *BMC Pregnancy Childbirth* 2016;**16**:1–12. doi:10.1186/s12884-016-1141-3

37 Seto TL, Tabangin ME, Josyula S, *et al.* Educational outcomes of Helping Babies Breathe training at a community hospital in Honduras. *Perspect Med Educ* 2015;**4**:225–32. doi:10.1007/s40037-015-0214-8

Confidential: For Review Only

Figure legends

Figure 1. Study selection process.

Figure 2. The duration of ventilation and interruptions in ventilation (seconds) during first minute after initiating positive pressure ventilation in the 99 resuscitations.

Figure 3. Cause of interruptions during first 2 minutes of initiated positive pressure ventilation.

Confidential: For Review Only

Confidential: For Review Only

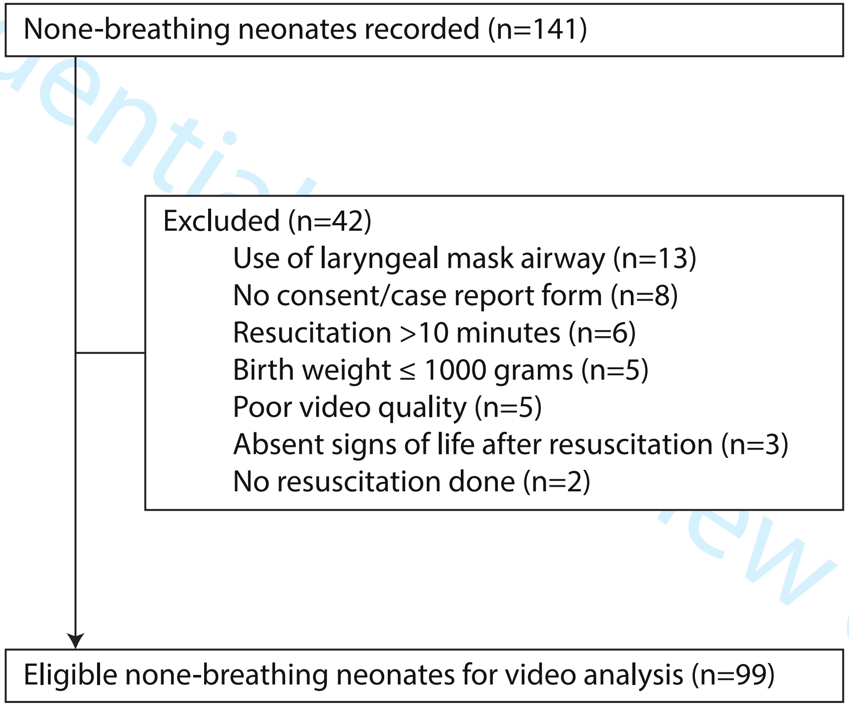

Confidential: For Review Only

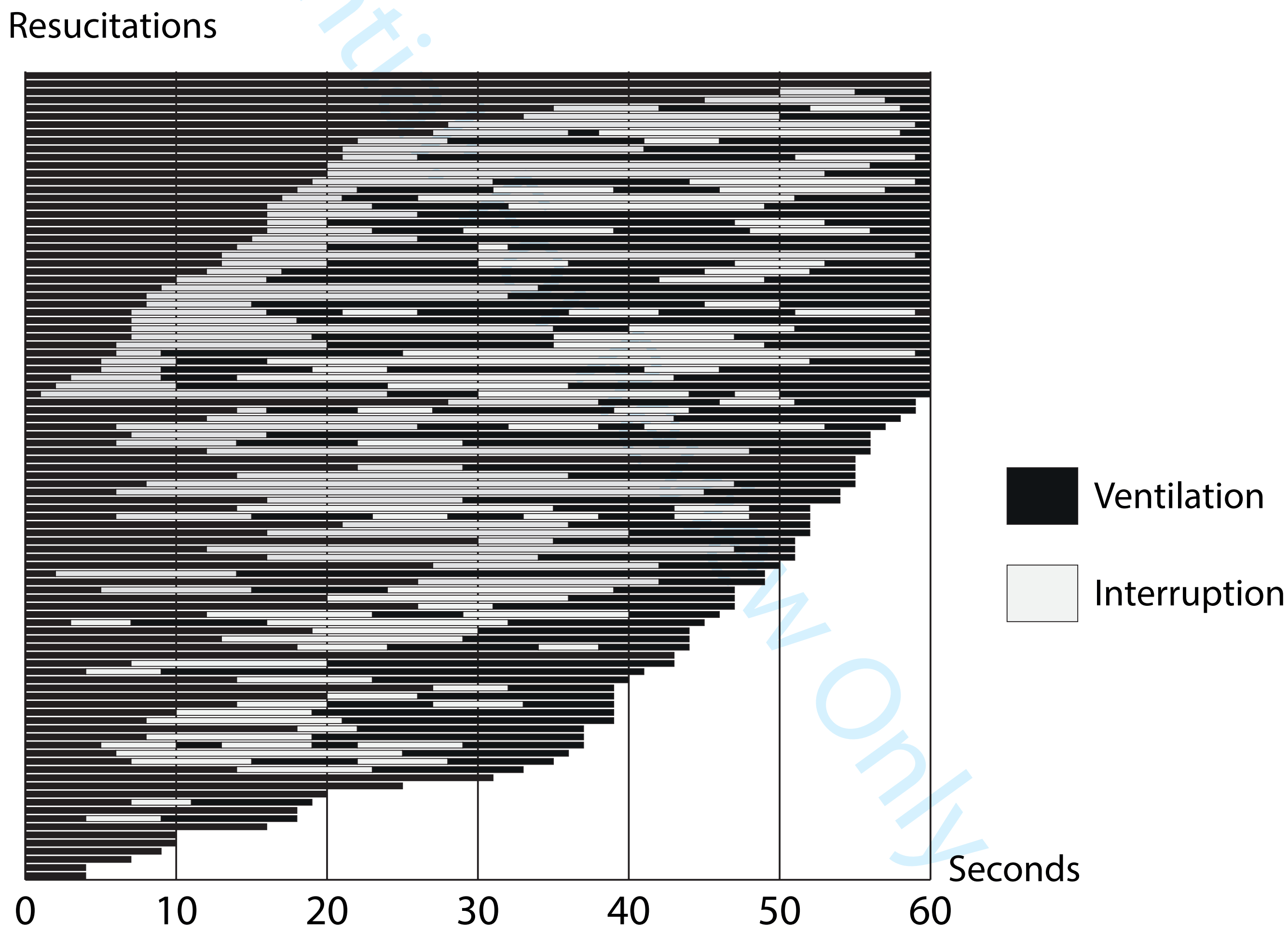

Confidential: For Review Only

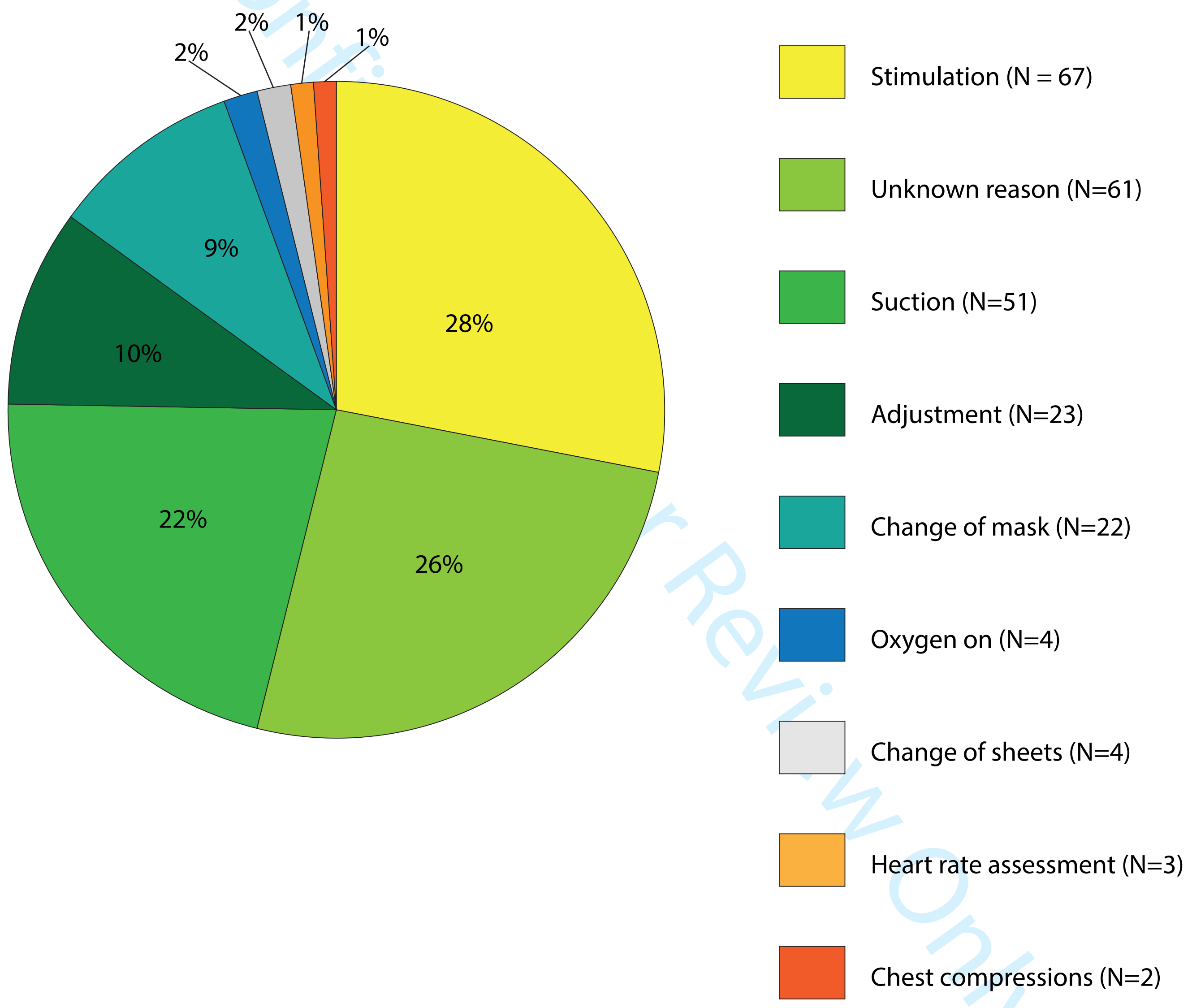

Confidential: For Review Only

BMJ Paediatrics Open

Neonatal resuscitation practices in Uganda - a video-observational study

Journal:	*BMJ Paediatrics Open*
Manuscript ID	bmjpo-2021-001092.R1
Article Type:	Original research
Date Submitted by the Author:	10-Jun-2021
Complete List of Authors:	Helldén, Daniel ; Karolinska Institute, Global Public Health<br>Myrnerts Höök, Susanna; Karolinska Institute, Global Public Health; University of Bergen Faculty of Medicine and Dentistry, Center for International Health<br>Pejovic, Nicolas; Karolinska Institute, Global Public Health; Sachs' Children and Youth Hospital<br>Mclellan, Dan; Karolinska Institute, Global Public Health<br>Lubulwa, Clare; Mulago Specialized Women and Neonatal Hospital<br>Tylleskär, Thorkild; University of Bergen Faculty of Medicine and Dentistry, Centre for International Health; University of Bergen, Centre for Intervention Science in Maternal and Child Health, Department of Global Public Health and Primary Care<br>Alfven, Tobias; Department of Global Public Health; Sachs' Children and Youth Hospital,
Keywords:	Neonatology, Resuscitation, Health services research

SCHOLARONE™
Manuscripts

I, the Submitting Author has the right to grant and does grant on behalf of all authors of the Work (as defined in the below author licence), an exclusive licence and/or a non-exclusive licence for contributions from authors who are: i) UK Crown employees; ii) where BMJ has agreed a CC-BY licence shall apply, and/or iii) in accordance with the terms applicable for US Federal Government officers or employees acting as part of their official duties; on a worldwide, perpetual, irrevocable, royalty-free basis to BMJ Publishing Group Ltd ("BMJ") its licensees and where the relevant Journal is co-owned by BMJ to the co-owners of the Journal, to publish the Work in this journal and any other BMJ products and to exploit all rights, as set out in our licence.

The Submitting Author accepts and understands that any supply made under these terms is made by BMJ to the Submitting Author unless you are acting as an employee on behalf of your employer or a postgraduate student of an affiliated institution which is paying any applicable article publishing charge ("APC") for Open Access articles. Where the Submitting Author wishes to make the Work available on an Open Access basis (and intends to pay the relevant APC), the terms of reuse of such Open Access shall be governed by a Creative Commons licence – details of these licences and which Creative Commons licence will apply to this Work are set out in our licence referred to above.

Other than as permitted in any relevant BMJ Author's Self Archiving Policies, I confirm this Work has not been accepted for publication elsewhere, is not being considered for publication elsewhere and does not duplicate material already published. I confirm all authors consent to publication of this Work and authorise the granting of this licence.

Confidential: For Review Only

Title: Neonatal resuscitation practices in Uganda - a video-observational study

Authors: Daniel Helldén[1]*, Susanna Myrnerts Höök*[1,2,3], Nicolas Pejovic[1,2,3], Dan McLellan[1], Clare Lubulwa[4], Thorkild Tylleskär[3,5], Tobias Alfvén[1,2]

Affiliations:

[1]Department of Global Public Health, Karolinska Institutet, Stockholm, Sweden

[2]Sachs' Children and Youth Hospital, Stockholm, Sweden

[3]Centre for International Health, University of Bergen, Bergen, Norway

[4]Mulago Specialized Women and Neonatal Hospital, Kampala, Uganda

[5]Center for Intervention Science in Maternal and Child Health, University of Bergen, Bergen, Norway

* These authors contributed equally

Keywords: Neonatal resuscitation, positive pressure ventilation, Helping Babies Breathe

Word count

Text: 2498

[1] Centre for International Health, University of Bergen, Bergen, Norway
Address: Postboks 7804, NO-5020 Bergen, Norway
Email: susanna.myrnerts-hook@sll.se
Telephone: +46737034362

Confidential: For Review Only

ABSTRACT

Background: Neonatal mortality, often due to birth asphyxia, remains stubbornly high in sub-Saharan Africa. Guidelines for neonatal resuscitation, where achieving adequate positive-pressure ventilation (PPV) is key, has been implemented in low-resource settings. However, the actual clinical practices of neonatal resuscitation have rarely been examined in these settings. The primary aim of this prospective observational study was to detail the cumulative proportion of time with ventilation during the first minute on the resuscitation table of neonates needing PPV at the Mulago National Referral Hospital in Kampala, Uganda.

Methods: From November 2015 to January 2016, resuscitations of non-breathing neonates by birth attendants were video-recorded using motion-sensor cameras. The resuscitation practices were analysed using the application NeoTapAS and compared between those taking place in the labour ward versus theatre through Fisher's exact test and Wilcoxon rank sum test.

Results: From 141 recorded resuscitations, 99 were included for analysis. The time to initiation of PPV were 66 (42-102) seconds overall, and there were minimal PPV during the first minute in both groups with 0 (0-10) and 0 (0-12) seconds of PPV, respectively. After initiating PPV the overall duration of interruptions during the first minute was 28 (18-37) seconds. The majority of interruptions were caused by stimulation (28%), unknown reasons (25%) and suction (22%).

Conclusions: Our findings show a low adherence to standard resuscitation practices in 2015-2016. This emphasizes the need for continuous educational efforts and investments in staff and adequate resources to increase the quality of clinical neonatal resuscitation practices in low-resource settings.

What is known about the subject?

- Timely initiation and duration of adequate ventilation is key to high-quality neonatal resuscitation

- Educational programs such as Helping Babies Breathe have been implemented to improve clinical practice in neonatal resuscitation

What this study adds?

- Analysis of video-recordings of actual neonatal resuscitation practices shows that they suffer from inadequate initiation and duration of positive-pressure ventilation, tendency for overstimulation, and excessive focus on suction.

- There is a strong need for continuous educational efforts and investments in staff and resources to increase quality of neonatal resuscitation practices in low-resource settings.

https://mc.manuscriptcentral.com/bmjpo

Confidential: For Review Only

INTRODUCTION

Intrapartum-related events (birth asphyxia) is the second leading cause of neonatal mortality and is estimated to cause approximately 700 000 deaths annually [1,2]. Neonatal mortality has declined at a slower pace in sub-Saharan Africa than in the rest of the world. In Uganda the neonatal mortality rate fell from 39 deaths per 1000 live births in 1990 to 20 in 2019 [3]. The country is currently not on track to meet the Sustainable Development Goal 3.2 stating that each country should aim for a neonatal mortality rate below 12 by 2030 [4]. Helping Babies Breathe (HBB) [5] is a basic neonatal resuscitation curriculum for low-resource settings aiming to improve skilled attendance at birth. National roll-out of HBB has been one of the cornerstones to further reduce the neonatal mortality [6,7].

Although the neonate´s transition from oxygenation from the placenta to pulmonary-based oxygenation is an intricate orchestration involving many aspects, for most neonates the process is uncomplicated [8]. Of all neonates born in the world annually, around 5-6 percent (7-9 million) will need neonatal resuscitation at birth [9]. High quality resuscitation has the potential to prevent neonatal deaths and complications from birth asphyxia. Positive-pressure ventilation (PPV) to ensure inflation of lungs and onset of alveolar aeration is key to successful resuscitation [10]. According to international guidelines, PPV should be initiated within one minute from birth and heart rate assessed after 30-60 seconds [11,12].

Video recordings have been suggested to be a valuable tool to evaluate resuscitation performance during neonatal resuscitations [13,14]. Difficulties to properly perform PPV have been reported even when highly experienced neonatologists resuscitate neonates [15,16]. In low-resource settings, clinical practices of neonatal resuscitation have rarely been examined. In this prospective observational study, we aimed to detail the crucial events of neonatal resuscitation performed by HBB-trained birth attendants in a low-resource setting. Our primary objective was to investigate the cumulative proportion of ventilation time during the first minute in neonates in need of PPV. Secondary objectives included examining interruptions in PPV and the cause for the interruptions.

METHODS

Study design

This observational study was performed utilizing video recordings of neonatal resuscitations at Mulago National Referral Hospital from 10th November 2015 to 26th January 2016. The sample was limited to the neonatal resuscitation attempts recorded during the study period for practical reasons, and given similar studies, a sample size of 140 resuscitation attempts were considered acceptable. Inclusion criteria were neonates needing PPV support taken to the resuscitation table where birth attendants, HBB trained or certified by similar neonatal resuscitation programmes, initiated the resuscitation. Exclusion criteria were recordings of resuscitations with bad video quality, recordings where no resuscitation was done, recordings were there were no apparent signs of life after resuscitation, resuscitations using laryngeal

Confidential: For Review Only

mask airway and resuscitations of neonates weighing ≤1000 grams. We also excluded the resuscitations where advanced resuscitation practices were initiated immediately upon arrival on table. Cases missing a report form or a video recording were also excluded.

Study setting

The Mulago National Referral Hospital is located in the capital city Kampala. The study was conducted at the high-risk labour ward and operating theatre, Department of Obstetrics and Gynaecology. The labour ward had 16 beds with one resuscitation table, and approximately 40-60 vaginal or assisted deliveries daily. On average, 15-20 caesarean sections were carried out per day in the two theatres, each with a neonatal resuscitation table. All three resuscitation tables were equipped with basic resuscitation equipment including 250 ml self-inflating bags and masks of size 0 and 1 for PPV as well as nasal cannulas with oxygen and bulb syringes for suction. Facemask was the most used tool used for PPV, while laryngeal mask airway was sometimes used.

Patient and public involvement

It was deemed not appropriate to involve patients or the public in the design, or conduct, or reporting or dissemination plans for the study.

Study procedures and data collection

All neonates needing PPV placed on the open resuscitation table during the study period were video recorded and assessed for eligibility. The research team estimated the time from birth to arrival to the resuscitation table to a mean time of 90 seconds in the labour ward and 30 seconds in theatre after observations and discussions with local birth attendants and hospital officials. The cameras (D-Link DCS 820L) were equipped with motion detection and started recording when a neonate was brought to the resuscitation table. Only the neonate and hands of the birth attendants were visible. The data from the cameras were downloaded and stored on securely kept external hard drives.

The free-of-charge iPad application NeoTapAS (Advanced Support) [17] was used to log and time the various events observed in the video recordings. There are two versions one for smartphones (NeoTapLS) and one for tablets (NeoTapAS). Studies have shown NeoTap to be a reliable, accurate and fast aid in assessing neonatal resuscitations [18–21]. Two investigators (DH and DM) reviewed and logged times and events for each included resuscitation. This included the duration of time of PPV, stimulation and suction. An interruption in ventilation was any type of activity that was not PPV after the PPV had started, for example stimulation or suction. A suction event was defined as when the bulb syringe was inserted in a nostril or the mouth and then removed. The head and mask position during PPV were observed, and if not the recommended neutral or sniffing position, or accurate mask placement described in the HBB were present this was noted together with if any type of heart assessment were made. If discrepancies arouse between the two logs, the first investigator (DH) reviewed the

Confidential: For Review Only

resuscitation again and made a final decision. This occurred for 25 resuscitations. From the NeoTapAS application the data were transferred to R [22] for statistical analysis.

Written and informed consent was obtained from birth attendants working at the labour ward and theatre during the study period. Qualifications, neonatal resuscitation training and whether they used the HBB action plan during their daily practice were noted. Deferred consent was obtained from the mothers of neonates being resuscitated before storing or viewing the corresponding video recording. A case report form containing the neonate's time of birth, mode of delivery, who delivered the baby, birth weight, gender, birth type (singleton or twin), Apgar score at 1 and 5 minutes, any abnormal features, the mother's socio-demographics, antenatal and natal history was filled in after the resuscitation if deferred consent was obtained.

Statistical analysis

Descriptive information regarding study selection process is presented along with descriptive statistics of mothers, neonates and the birth attendants in numbers (%). Due to the different locations and modes of birth, resuscitations taking place in the labour ward were compared with resuscitations taking place in theatre. The primary and secondary outcomes were analysed through descriptive statistics with numbers (%) for categorial variables and median (interquartile range) and compared through Fisher's exact test and Wilcoxon rank sum test due to the dependent variable having low number of counts and being non-parametric. All quantitative data were analysed in R [22]. A p-value <0.05 was considered to be statistically significant.

RESULTS

In total 141 resuscitation attempts were recorded, of these 42 were excluded primarily due to use of laryngeal mask airway and no consent or case report form, which resulted in 99 video recordings eligible for timing and logging (**Figure 1**).

Baseline characteristics

Pregnancy complications were common (**Table 1**). Vaginal and caesarean delivery mode ratio was 1:1. The median number of neonates with Apgar score 0-3 at minute 1 and minute 5 was 28 (28%) and 6 (6%), respectively. A shift in overall median Apgar score between first and fifth minute from 4-6 to ≥7 was observed. Most neonates weighed ≥3000 grams. All birth attendants had at least a midwifery certificate and all but 4 birth attendants claimed to use the HBB action plan during resuscitations.

https://mc.manuscriptcentral.com/bmjpo

Confidential: For Review Only

Table 1. Characteristics of included mothers, neonates and birth attendants.

**Mothers**	Number N = 99 n (%)	**Neonates**	Number N = 99 n (%)	**Birth attendants**	Number N = 45 n (%)
**Age (years)**		**Gender**		**Age (years)**	
15 – 19	15 (15)	Female	52 (52)	20 – 30	14 (31)
20 - 24	42 (42)	Male	47 (47)	31 – 41	21 (47)
30 – 34	10 (10)	**Birth type**		42 – 52	10 (23)
35 – 49	11 (11)	Singleton	90 (90)	**Qualification level**	
**Antenatal attendance**		Twins	9 (9)	Degree nurse	2 (4)
Yes	96 (97)	**Apgar score at 1 minute**		Diploma midwife	27 (60)
No	3 (3)	0 – 3	28 (28)	Certificate midwife	16 (36)
**Birth order**		4 – 6	58 (58)	**Attended HBB refresher course**	
First child	38 (38)	≥ 7	12 (12)	Yes	27 (60)
Second child	19 (19)	**Apgar score at 5 minutes[2]**		No	18 (40)
≥ Third child	42 (42)	0 – 3	6 (6)	**Time since last HBB refresher course**	
**Pregnancy complications**		4 – 6	45 (45)	≤ 6 months	10 (37)
Obstructed labour	14 (14)	≥ 7	48 (48)	6 – 12 months	4 (15)
Fetal distress	4 (4)	**Birth weight in grams**		≥ 12 months	13 (48)
Pre-eclampsia/eclampsia	3 (3)	1000 – 1499	7 (7)	**Reasons for not attending HBB refresher course[3]**	
Breech	5 (5)	1500 – 1999	9 (9)	I have adequate knowledge	8 (18)
Cord prolapse	2 (2)	2000 – 2999	26 (26)	No time	9 (20)
Oligohydramnios	6 (6)	≥ 3000	56 (56)	No finances	10 (22)
Preterm birth	1 (1)			No such course was offered	4 (9)
Other[1]	16 (16)			No opportunity	5 (11)
No complications	48 (48)			No answer	2 (4)
**Mode of delivery**				**Used the HBB action plan during resuscitations**	
Spontaneous vaginal delivery	48 (48)			Yes	41 (91)
Caesarean section	48 (48)			No	4 (9)
Instrumental delivery	3 (3)				

[1]Other: cervical dystocia, bleeding conditions such as placenta previa or placenta abruption, delayed second stage of labour which in 14 out of 16 occurrences resulted in a caesarean section. [2]Apgar score at 10 minutes was not included as most attention is given to the scoring at 5 minutes.

Ventilation

The median (IQR) time from birth to initiation of PPV was 137 (94-168) seconds, and from arrival to table 66 (44-102) seconds (**Table 2**). There was minimal PPV during minute 1 after arrival to the resuscitation table. In resuscitations taking place in the labour ward there were 0 (0-10) seconds of PPV and 0 (0-12) seconds in the theatre, and during minute 2 resuscitations taking place in the labour ward included 19 (7-32) seconds of PPV while resuscitations in the theatre had 18 (8-25) seconds of ventilation. Overall, during the first 2 minutes after arriving on the table there had only been 21 (12-36) seconds of PPV with no significant difference between the place of birth. Additionally, approximately a third of the

Confidential: For Review Only

neonates did not have the recommended neutral to sniffing head position during ventilation and the resuscitation was mainly performed by a single birth attendant. The duration of interruptions in ventilations during minute 1 after initiating PPV for all resuscitations was 25 (15-35) and 29 (24-38) seconds of interruption, respectively, **Figure 2** and **Table 2**. Interruptions persisted, and for the first 2 minutes after initiating PPV there were 72 (47-89) seconds of interruption for both groups. Stimulation 28% (n=67), no apparent reason 26% (n=61) and suction 22% (n=51) were the primary causes for interruptions, while corrective adjustments of the position of the neonate and change of facemask were also common causes (**Figure 3**).

Table 2. Resuscitation procedures during first and second minute after arriving at the resuscitation table.

**Place of birth**	Total (N= 99)	Labour ward (N= 51)	Theatre (N=48)	p-value
**Time**	**Seconds (IQR)**	**Seconds (IQR)**	**Seconds (IQR)**	
**Ventilation**				
Time to initiation of PPV from birth[1]	137 (94-168)	161 (142-197)	92 (68-113)	<0.001
Time to initiation of PPV after arrival on table	66 (44-102)	71 (52-107)	62 (38-83)	0.126
Total duration of PPV minute 1	0 (0-10)	0 (0-10)	0 (0-12)	0.159
Total duration of PPV minute 2	18 (7-30)	19 (7-32)	18 (8-25)	0.623
Total duration of PPV first 2 minutes	21 (12-36)	19 (9-43)	23 (14-32)	0.616
Total duration of interruption minute 1 of ventilation	28 (18-37)	25 (15-35)	29 (24-38)	0.132
Total duration of interruption first 2 minutes of ventilation	72 (47-89)	68 (45-84)	81 (57-93)	0.059
**Stimulation**				
Total stimulation time minute 1	9 (3-18)	5 (0-9)	16 (11-21)	<0.001
Total stimulation time minute 2	5 (0-10)	4 (0-8)	2 (6-10)	0.125
Total stimulation time first 2 minutes	16 (7-26)	8 (4-21)	24 (15-29)	<0.001
**Suction**				
Total suction time minute 1	21 (8-29)	23 (10-33)	18 (6-25)	0.136
Total suction time minute 2	7 (0-20)	4 (0-21)	9 (0-19)	0.244
Total suction time first 2 minutes	28 (15-43)	28 (16-44)	29 (14-43)	0.737
**Frequency**	**Number (%)**	**Number (%)**	**Number (%)**	
Only one birth attendant	62 (63)	29 (57)	33 (69)	0.299
**Ventilation procedure**				
Not recommended head position	31 (31)	8 (16)	23 (48)	0.001
Not accurate mask position	16 (16)	5 (10)	11 (23)	0.103
**Heart rate assessment**				
By auscultation	1 (1)	1 (2)	0 (0)	1
By umbilical cord palpation	5 (5)	1 (2)	4 (8)	0.196
By chest palpation	11 (11)	9 (18)	2 (4)	0.052

Results are presented as median seconds (IQR) or numbers (%) and compared with Wilcoxon rank sum test for continuous and Fisher's exact test for categorical variables. [1] For resuscitations taking

Confidential: For Review Only

place at the labour ward (n=51) and theatre (n=48) the estimated time from birth to resuscitation was 90 seconds and 30 seconds respectively. All other measurements in the table are from when the neonate arrives on the resuscitation table.

Stimulation and suction

Stimulation time during minute 1 was significantly lower for the resuscitations taking place in the labour ward 5 (0-9) versus 16 (11-21) seconds (p-value <0.001), stimulation was less present in minute 2 (**Table 2**). Overall, for the first 2 minutes after arriving to the table the duration of stimulation was 16 (7-26) seconds. Suction was prevalent for the whole resuscitation but particularly during minute 1, the cumulative suction time for both groups was 21 (8-29) seconds with a median of 5 (2-8) suction events with bulb syringes per neonate. During minute 2 there was less duration of suction in both groups. A median of 8 (4-13) separate suction events per neonate which led to a cumulative time effort of 28 (15-43) seconds were recorded during the first 2 minutes after arriving to the resuscitation table. Lastly, only 17% of the resuscitations included any form of heart rate assessment.

DISCUSSION

This observational study used video recordings of neonatal resuscitations in a country with high neonatal mortality. Our findings demonstrate a low adherence to standard resuscitation practices in 2015-2016 with inadequate initiation and duration of PPV, tendency to overstimulate, and excessive focus on suction. This adds to the comparable resuscitation patterns with insufficient or incorrect ventilation efforts found in a similar setting in Mozambique [23], while a study in Nepal concluded that none of the examined resuscitations met the golden minute guideline standard [24]. However, failing to initiate proper ventilation within the first minute and applying suboptimal PPV is also common in high-resource settings [25–27]. Specifically, a study from a tertiary hospital in Norway report a median time from arriving to the table to initiation of PPV of 42 seconds and 56% of neonates received PPV with a 60% ventilation fraction during the first 30 seconds. [25] The time to initiation of PPV was considerably longer in our study and does not commence within the recommended golden minute after birth. However, in general it seems that ventilation during resuscitations tend not to be commenced within the recommended time in both high- and low-resource settings.

In our study, the high number and relatively long duration of interruptions was reflected in the low ventilation time during the first minute of ventilation not meeting the recommended 60 seconds of continuous PPV before assessing the adequacy of ventilation. We build on the previous literature by showcasing what the actual causes of those interruptions were. In our study, most interruptions were due to stimulation, unknown reasons, and suctioning, which should not disrupt PPV. In the Norwegian study [25] the main reasons for ventilation interruptions were for adjustments for optimising ventilation, heart rate evaluation, and stimulation. This was often not the case in our study. It is, however, important to acknowledge and stress the difference in staff quantity and resources between these two settings. According to a qualitative study from Tanzania the main reasons, as per the birth

Confidential: For Review Only

attendants themselves, for delay or interruptions of PPV were fear of doing a poor job in an acute situation, difficulties in assessing the neonate and in taking appropriate action [28]. Similar findings have been reported from high-resource settings as well [29]. It is highly likely that the lack of adherence to guidelines in our study stems from both a lack of knowledge and professional confidence, compounded by the limited resources and low number of staff.

Our study reveals excessive amounts of both stimulation and suctioning, the former being the main reason for ventilation interruptions adding strength to similar findings in other settings [25,30,31]. Intriguingly, Wrammert et al. [32] showed excessive usage of both stimulation and suctioning as main reasons to ventilation delay and halt in resuscitations conducted in Nepal, and proposed that the continuation of the phenomenon was due to difficulties of abandoning a tradition of suctioning of non-breathing neonates before the implementation of HBB or similar educational programs. Further, it is understandable that in an acute situation where time is of the essence, one might want to utilize other strategies if ventilation seems fruitless. Excessive usage of stimulation and suctioning in our study could be explained by this need, requiring special attention when developing educational programs. Lastly, heart rate assessments were rarely done, a vital component of neonatal resuscitation often underused in low-resource settings [33].

Almost all birth attendants claimed to be using the HBB action plan routinely when working with neonatal resuscitation. The results in our study however display a real-world situation where set guidelines were not followed. The need for and beneficial effects of acquired skills and knowledge to be frequently updated, practiced and evaluated is clear while similarly important is to increase staff numbers and forming close working professional teams for resuscitations [24,34–36].

Strengths and weaknesses

Most limitations of this study are inherent to post-hoc video analysis. First, the factual time spent between birth and arrival to table was estimated and may not reflect the correct time in each case. Second, assessing recorded resuscitation procedures relies on the clinical expertise of the reviewer, and distinguishing between different procedures is not always clear. Third, though an improvement in Apgar scores from minute 1 to minute 5 was observed, it was not possible to evaluate the effectiveness of PPV since no certain observation of tidal volumes or pulse oximetry was performed. However, by using video recordings the most critical aspects of actual resuscitation practices can be examined and timely logged, and an overall picture formed.

Conclusion

This study demonstrates real life neonatal resuscitation practices in 2015-2016 that are not in line with guidelines with inadequate initiation and duration of PPV, tendency for overstimulation and excessive focus on suction. There is a strong need for continuous

Confidential: For Review Only

educational efforts and investments in adequate resources to increase the quality of clinical neonatal resuscitation practices in low-resource settings.

Acknowledgements

We would like to give thanks to all participants of this study. Our greatest thanks to the staff at the Maternity and Special Care Unit of Mulago National Referral Hospital, Kampala, Uganda, for their continuous struggle to improve the care of neonates.

Contributors

DH was responsible for data analysis, interpretation of data and for the writing process including writing a first draft of the manuscript and approval of the final draft. SMH was responsible for the study design and acquisition of data from video recordings. She revised the manuscript and approved the final draft. NJP contributed to study design and acquisition of data. He revised the manuscript and approved the final draft. DM assisted in data analysis and writing process, revised the manuscript and approved the final draft. CL contributed to study design, was responsible for preparation of the study site and collecting the video recordings. She revised the manuscript and approved the final draft. TT contributed to study design, interpretation of data, manuscript revision and approved the final draft. TA contributed to the study design, data analysis, manuscript revision and approval of the final draft.

Funding

The study was funded by the Norwegian Government through the Norwegian Quota Scholarship scheme and by in-kind contributions from the University of Bergen, Norway, Makerere University, Uganda, and the Karolinska Institutet, Sweden.

Competing interests

The authors have no financial relationships relevant to this article to disclose. SMH, NJP, CL, TT and TA are co-founders of the non-profit organisation Tap4Life (www.tap4life.org), which produced the free-of-charge application NeoTap. The SMH, NJP, CL, TT and TA do not get any salary from Tap4Life.

Patient consent for publication

Not required.

Ethical approval

Ethical approval of the study was received from the Mulago Hospital Research and Ethics Committee and the Uganda National Council of Science and Technology (MREC:841). The Norwegian research ethics committee exempted it from evaluation since it was deemed a quality assurance study.

Confidential: For Review Only

Data availability statement

All data relevant to this study are included in the article.

References

1 Ehret DY, Patterson JK, Bose CL. Improving Neonatal Care: A Global Perspective. Clin. Perinatol. 2017;**44**:567–82. doi:10.1016/j.clp.2017.05.002

2 Liu L, Oza S, Hogan D, *et al.* Global, regional, and national causes of child mortality in 2000-13, with projections to inform post-2015 priorities: an updated systematic analysis. *Lancet* 2015;**385**:430–40. doi:10.1016/S0140-6736(14)61698-6

3 UN Inter-agency Group for Child Mortality Estimation. Levels & Trend in Child Mortality: Report 2020. New York: 2020. https://www.unicef.org/media/79371/file/UN-IGME-child-mortality-report-2020.pdf.pdf

4 Hug L, Alexander M, You D, *et al.* National, regional, and global levels and trends in neonatal mortality between 1990 and 2017, with scenario-based projections to 2030: a systematic analysis. *Lancet Glob Heal* 2019;**7**:e710–20. doi:10.1016/S2214-109X(19)30163-9

5 Kamath-Rayne BD, Thukral A, Visick MK, *et al.* Helping Babies Breathe, second edition: A model for strengthening educational programs to increase global newborn survival. *Glob Heal Sci Pract* 2018;**6**:538–51. doi:10.9745/GHSP-D-18-00147

6 Reisman J, Arlington L, Jensen L, *et al.* Newborn resuscitation training in resource-limited settings: A systematic literature review. Pediatrics. 2016;**138**. doi:10.1542/peds.2015-4490

7 Kamath-Rayne BD, Berkelhamer SK, Kc A, *et al.* Neonatal resuscitation in global health settings: An examination of the past to prepare for the future. *Pediatr Res* 2017;**82**:194–200. doi:10.1038/pr.2017.48

8 Morton SU, Brodsky D. Fetal Physiology and the Transition to Extrauterine Life. Clin. Perinatol. 2016;**43**:395–407. doi:10.1016/j.clp.2016.04.001

9 UNICEF. State of the World's Children 2019: Children, food and nutrition. New York: 2019. https://www.unicef.org/reports/state-of-worlds-children-2019

10 Hooper SB, Siew ML, Kitchen MJ, *et al.* Establishing functional residual capacity in the non-breathing infant. Semin. Fetal Neonatal Med. 2013;**18**:336–43. doi:10.1016/j.siny.2013.08.011

11 Perlman JM, Wyllie J, Kattwinkel J, *et al.* Part 7: Neonatal resuscitation 2015 international consensus on cardiopulmonary resuscitation and emergency cardiovascular care science with treatment recommendations. *Pediatrics* 2015;**136**:S120–66. doi:10.1542/peds.2015-3373D

Confidential: For Review Only

12 World Health Organization. Guidlines on basic newborn resuscitation. Geneva: 2012. https://apps.who.int/iris/bitstream/handle/10665/75157/9789241503693_eng.pdf?sequence=1

13 Carbine DN, Finer NN, Knodel E, *et al.* Video Recording as a Means of Evaluating Neonatal Resuscitation Performance. *Pediatrics* 2000;**106**:654–8. doi:doi: 10.1542/peds.106.4.654

14 Schilleman K, Siew ML, Lopriore E, *et al.* Auditing resuscitation of preterm infants at birth by recording video and physiological parameters. *Resuscitation* 2012;**83**:1135–9. doi:10.1016/j.resuscitation.2012.01.036

15 Schmölzer GM, Dawson JA, Kamlin COF, *et al.* Airway obstruction and gas leak during mask ventilation of preterm infants in the delivery room. *Arch Dis Child Fetal Neonatal Ed* 2011;**96**:F254-7. doi:10.1136/adc.2010.191171

16 Moshiro R, Perlman JM, Kidanto H, *et al.* Predictors of death including quality of positive pressure ventilation during newborn resuscitation and the relationship to outcome at seven days in a rural Tanzanian hospital. *PLoS One* 2018;**13**:e0202641. doi:10.1371/journal.pone.0202641

17 Tap4Life. http://tap4life.org/

18 Myrnerts Höök S, Pejovic NJ, Marrone G, *et al.* Accurate and fast neonatal heart rate assessment with a smartphone-based application – a manikin study. *Acta Paediatr Int J Paediatr* 2018;**107**:1548–54. doi:10.1111/apa.14350

19 Johnson PA, Morina N, O'Reilly M, *et al.* Evaluation of a Tap-Based Smartphone App for Heart Rate Assessment During Asphyxia in a Porcine Model of Neonatal Resuscitation. *Front Pediatr* 2019;**7**:453. doi:10.3389/fped.2019.00453

20 Binotti M, Cavallin F, Ingrassia PL, *et al.* Heart rate assessment using NeoTapAdvancedSupport: A simulation study. *Arch Dis Child Fetal Neonatal Ed* 2019;**104**:F440–2. doi:10.1136/archdischild-2018-315408

21 Myrnerts Höök S, Pejovic NJ, Cavallin F, *et al.* Smartphone app for neonatal heart rate assessment: an observational study. *BMJ Paediatr Open* 2020;**4**:e000688. doi:10.1136/bmjpo-2020-000688

22 R Core Team. R: A language and environment for statistical computing. 2019.https://www.r-project.org/

23 Trevisanuto D, Bertuola F, Lanzoni P, *et al.* Effect of a neonatal resuscitation course on healthcare providers' performances assessed by video recording in a low-resource setting. *PLoS One* 2015;**10**:1–12. doi:10.1371/journal.pone.0144443

24 Kc A, Wrammert J, Clark RB, *et al.* Reducing perinatal mortality in Nepal using helping babies breathe. *Pediatrics* 2016;**137**:e20150117. doi:10.1542/peds.2015-0117

25 Skåre C, Boldingh AM, Nakstad B, *et al.* Ventilation fraction during the first 30 s of neonatal resuscitation. *Resuscitation* 2016;**107**:25–30.

Confidential: For Review Only

doi:10.1016/j.resuscitation.2016.07.231

26 Bjorland PA, Ersdal HL, Øymar K, *et al.* Compliance with guidelines and efficacy of heart rate monitoring during newborn resuscitation: A prospective video study. *Neonatology* 2020;**117**:175–81. doi:10.1159/000506772

27 Niles DE, Cines C, Insley E, *et al.* Incidence and characteristics of positive pressure ventilation delivered to newborns in a US tertiary academic hospital. *Resuscitation* 2017;**115**:102–9. doi:10.1016/j.resuscitation.2017.03.035

28 Moshiro R, Ersdal HL, Mdoe P, *et al.* Factors affecting effective ventilation during newborn resuscitation: a qualitative study among midwives in rural Tanzania. *Glob Health Action* 2018;**11**:1423862. doi:10.1080/16549716.2018.1423862

29 Ljungblad LW, Skovdahl K, McCormack B, *et al.* Balancing life and death during the golden minute – midwives' experiences of performing newborn resuscitation. *J Multidiscip Healthc* 2020;**13**:943–52. doi:10.2147/JMDH.S268959

30 Bansal SC, Nimbalkar AS, Patel D V., *et al.* Current Neonatal Resuscitation Practices among Paediatricians in Gujarat, India. *Int J Pediatr* 2014;**2014**:1–7. doi:10.1155/2014/676374

31 Adams E, O´Neal P, Hanson L, *et al.* Airway clearance of the term newborn. *MCN Am J Matern Child Nurs* 2018;**43**:306–12. doi:10.1097/NMC.0000000000000473

32 Wrammert J, Zetterlund C, Ashish KC, *et al.* Resuscitation practices of low and normal birth weight infants in Nepal: An observational study using video camera recordings. *Glob Health Action* 2017;**10**:1322372. doi:10.1080/16549716.2017.1322372

33 Johnson PA, Schmölzer GM. Heart Rate Assessment during Neonatal Resuscitation. *Healthcare* 2020;**8**:43. doi:10.3390/healthcare8010043

34 Hodgins S. Helping babies breathe - beyond training. *Glob Heal Sci Pr* 2018;**6**:402–4. doi:10.1016/j.jpeds.2012.03.030

35 Bang A, Patel A, Bellad R, *et al.* Helping Babies Breathe (HBB) training: What happens to knowledge and skills over time? *BMC Pregnancy Childbirth* 2016;**16**:1–12. doi:10.1186/s12884-016-1141-3

36 Seto TL, Tabangin ME, Josyula S, *et al.* Educational outcomes of Helping Babies Breathe training at a community hospital in Honduras. *Perspect Med Educ* 2015;**4**:225–32. doi:10.1007/s40037-015-0214-8

Confidential: For Review Only

Figure legends

Figure 1. Study profile.

Figure 2. The duration of ventilation and interruptions in ventilation (seconds) during first minute after initiating positive pressure ventilation in the 99 resuscitations.

Figure 3. Cause of interruptions during first 2 minutes of initiated positive pressure ventilation.

Confidential: For Review Only

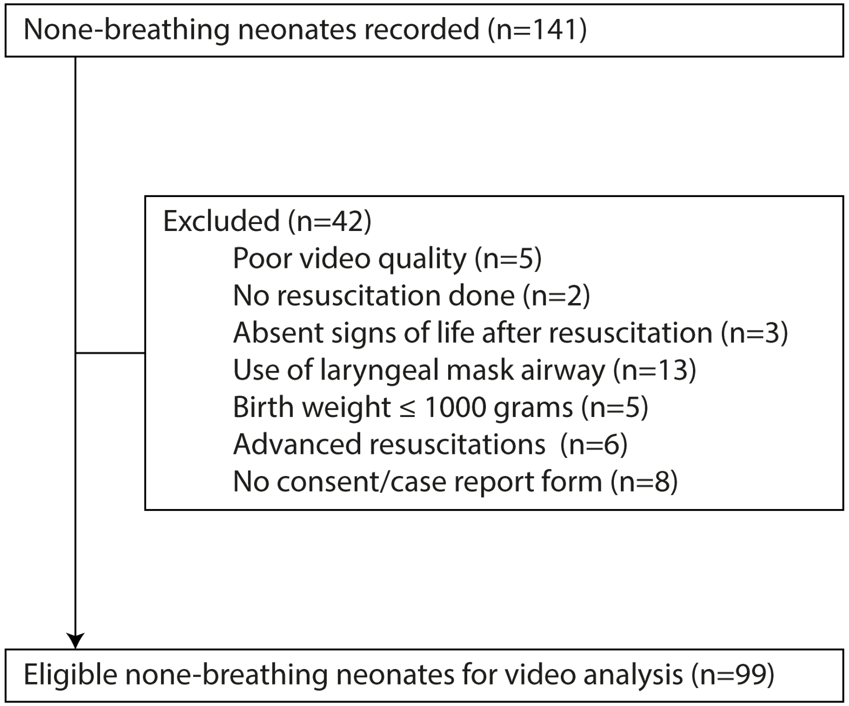
None-breathing neonates recorded (n=141)
Excluded (n=42)
Poor video quality (n=5)
No resuscitation done (n=2)
Absent signs of life after resuscitation (n=3)
Use of laryngeal mask airway (n=13)
Birth weight ≤ 1000 grams (n=5)
Advanced resuscitations (n=6)
No consent/case report form (n=8)
Eligible none-breathing neonates for video analysis (n=99)

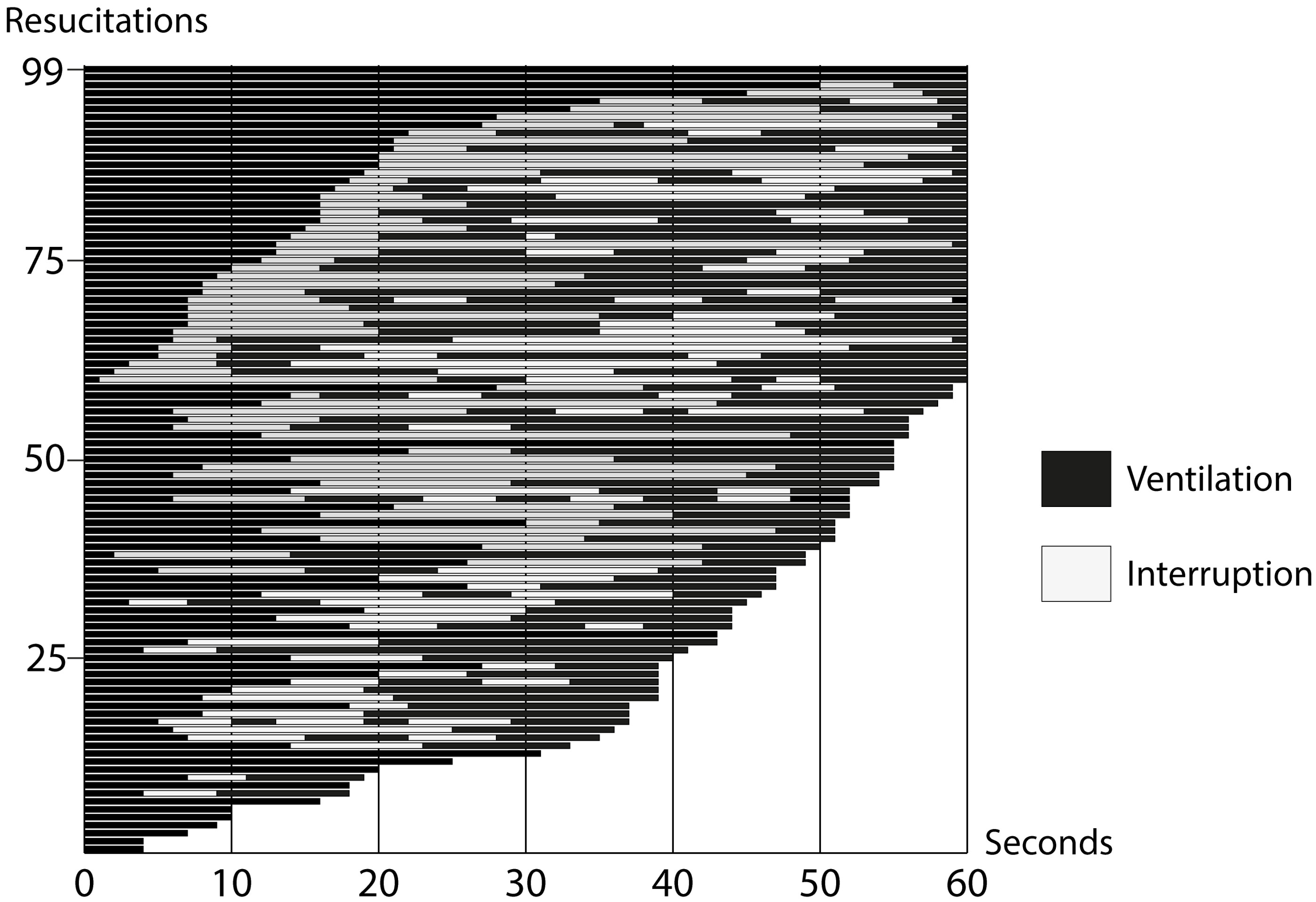
Resucitations
99
75
50
25
0
10
20
30
40
50
60
Seconds
Ventilation
Interruption

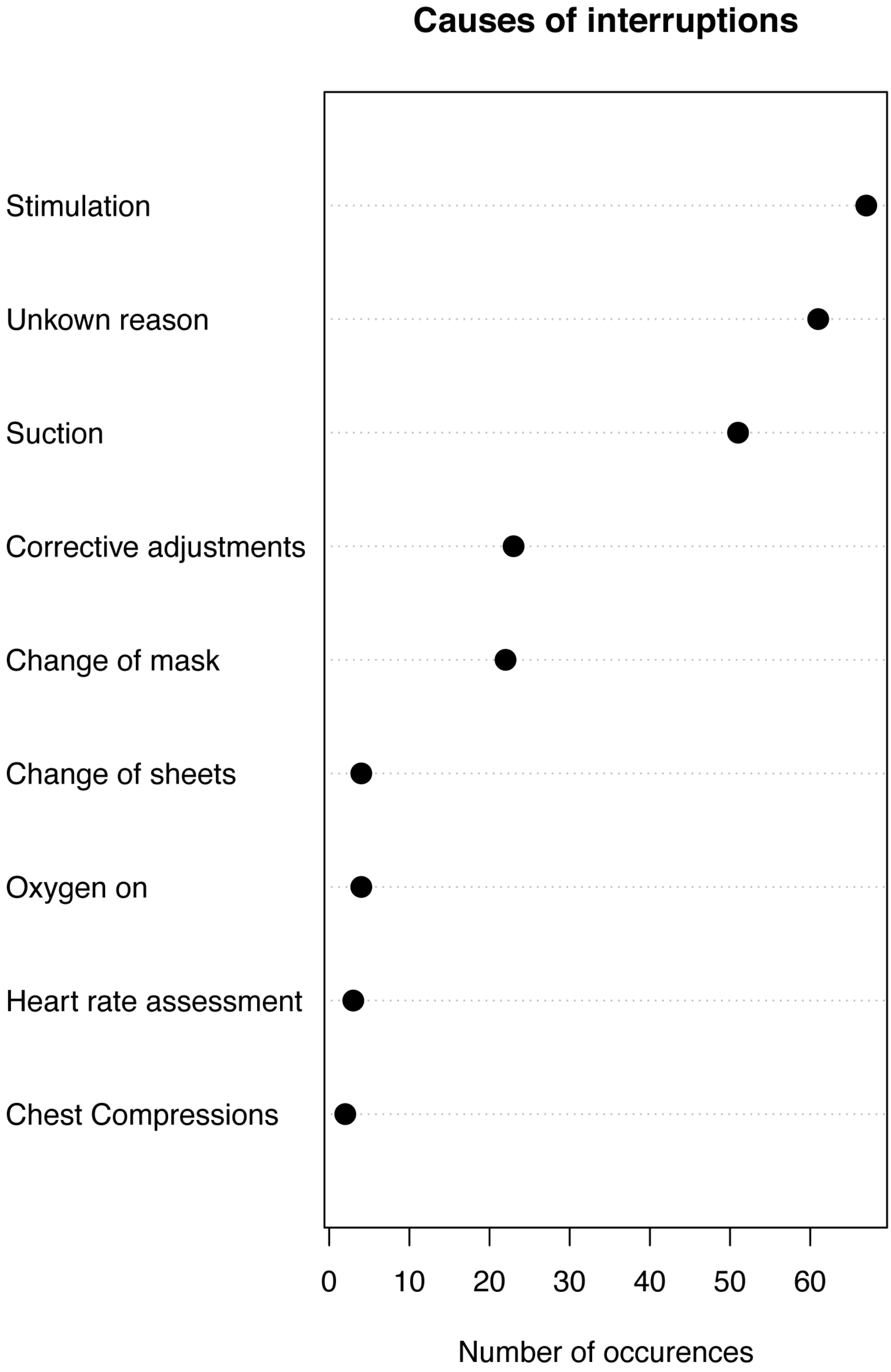
Causes of interruptions
Stimulation
Unkown reason
Suction
Corrective adjustments
Change of mask
Change of sheets
Oxygen on
Heart rate assessment
Chest Compressions
0
10
20
30
40
50
60
Number of occurences

BMJ Paediatrics Open

Neonatal resuscitation practices in Uganda - a video-observational study

Journal:	*BMJ Paediatrics Open*
Manuscript ID	bmjpo-2021-001092.R2
Article Type:	Original research
Date Submitted by the Author:	27-Jul-2021
Complete List of Authors:	Helldén, Daniel ; Karolinska Institute, Global Public Health<br>Myrnerts Höök, Susanna; Karolinska Institute, Global Public Health; University of Bergen Faculty of Medicine and Dentistry, Center for International Health<br>Pejovic, Nicolas; Karolinska Institute, Global Public Health; Sachs' Children and Youth Hospital<br>Mclellan, Dan; Karolinska Institute, Global Public Health<br>Lubulwa, Clare; Mulago Specialized Women and Neonatal Hospital<br>Tylleskär, Thorkild; University of Bergen Faculty of Medicine and Dentistry, Centre for International Health; University of Bergen, Centre for Intervention Science in Maternal and Child Health, Department of Global Public Health and Primary Care<br>Alfven, Tobias; Department of Global Public Health; Sachs' Children and Youth Hospital,
Keywords:	Neonatology, Resuscitation, Health services research

SCHOLARONE™
Manuscripts

I, the Submitting Author has the right to grant and does grant on behalf of all authors of the Work (as defined in the below author licence), an exclusive licence and/or a non-exclusive licence for contributions from authors who are: i) UK Crown employees; ii) where BMJ has agreed a CC-BY licence shall apply, and/or iii) in accordance with the terms applicable for US Federal Government officers or employees acting as part of their official duties; on a worldwide, perpetual, irrevocable, royalty-free basis to BMJ Publishing Group Ltd ("BMJ") its licensees and where the relevant Journal is co-owned by BMJ to the co-owners of the Journal, to publish the Work in this journal and any other BMJ products and to exploit all rights, as set out in our licence.

The Submitting Author accepts and understands that any supply made under these terms is made by BMJ to the Submitting Author unless you are acting as an employee on behalf of your employer or a postgraduate student of an affiliated institution which is paying any applicable article publishing charge ("APC") for Open Access articles. Where the Submitting Author wishes to make the Work available on an Open Access basis (and intends to pay the relevant APC), the terms of reuse of such Open Access shall be governed by a Creative Commons licence – details of these licences and which Creative Commons licence will apply to this Work are set out in our licence referred to above.

Other than as permitted in any relevant BMJ Author's Self Archiving Policies, I confirm this Work has not been accepted for publication elsewhere, is not being considered for publication elsewhere and does not duplicate material already published. I confirm all authors consent to publication of this Work and authorise the granting of this licence.

Confidential: For Review Only

Title: Neonatal resuscitation practices in Uganda - a video-observational study

Authors: Daniel Helldén[1]*, Susanna Myrnerts Höök[*1,2,3], Nicolas Pejovic[1,2,3], Dan McLellan[1], Clare Lubulwa[4], Thorkild Tylleskär[3,5], Tobias Alfvén[1,2]

Affiliations:

[1]Department of Global Public Health, Karolinska Institutet, Stockholm, Sweden

[2]Sachs' Children and Youth Hospital, Stockholm, Sweden

[3]Centre for International Health, University of Bergen, Bergen, Norway

[4]Mulago Specialized Women and Neonatal Hospital, Kampala, Uganda

[5]Center for Intervention Science in Maternal and Child Health, University of Bergen, Bergen, Norway

* These authors contributed equally

Keywords: Neonatal resuscitation, positive pressure ventilation, Helping Babies Breathe

Word count

Text: 2586

Confidential: For Review Only

[1] Centre for International Health, University of Bergen, Bergen, Norway
Address: Postboks 7804, NO-5020 Bergen, Norway
Email: susanna.myrnerts-hook@sll.se
Telephone: +46737034362

ABSTRACT

Background: Neonatal mortality, often due to birth asphyxia, remains stubbornly high in sub-Saharan Africa. Guidelines for neonatal resuscitation, where achieving adequate positive-pressure ventilation (PPV) is key, has been implemented in low-resource settings. However, the actual clinical practices of neonatal resuscitation have rarely been examined in these settings. The primary aim of this prospective observational study was to detail the cumulative proportion of time with ventilation during the first minute on the resuscitation table of neonates needing PPV at the Mulago National Referral Hospital in Kampala, Uganda.

Methods: From November 2015 to January 2016, resuscitations of non-breathing neonates by birth attendants were video-recorded using motion-sensor cameras. The resuscitation practices were analysed using the application NeoTapAS and compared between those taking place in the labour ward versus theatre through Fisher's exact test and Wilcoxon rank sum test.

Results: From 141 recorded resuscitations, 99 were included for analysis. The time to initiation of PPV were 66 (42-102) seconds overall, and there were minimal PPV during the first minute in both groups with 0 (0-10) and 0 (0-12) seconds of PPV, respectively. After initiating PPV the overall duration of interruptions during the first minute was 28 (18-37) seconds. The majority of interruptions were caused by stimulation (28%), unknown reasons (25%) and suction (22%).

Conclusions: Our findings show a low adherence to standard resuscitation practices in 2015-2016. This emphasizes the need for continuous educational efforts and investments in staff and adequate resources to increase the quality of clinical neonatal resuscitation practices in low-resource settings.

What is known about the subject?

- Timely initiation and duration of adequate ventilation is key to high-quality neonatal resuscitation

- Educational programs such as Helping Babies Breathe have been implemented to improve clinical practice in neonatal resuscitation

What this study adds?

- Analysis of video-recordings of actual neonatal resuscitation practices shows that they suffer from inadequate initiation and duration of positive-pressure ventilation, tendency for overstimulation, and excessive focus on suction.

- There is a strong need for continuous educational efforts and investments in staff and resources to increase quality of neonatal resuscitation practices in low-resource settings.

Confidential: For Review Only

INTRODUCTION

Intrapartum-related events (birth asphyxia) is the second leading cause of neonatal mortality and is estimated to cause approximately 700 000 deaths annually [1,2]. Neonatal mortality has declined at a slower pace in sub-Saharan Africa than in the rest of the world. In Uganda the neonatal mortality rate fell from 39 deaths per 1000 live births in 1990 to 20 in 2019 [3]. The country is currently not on track to meet the Sustainable Development Goal 3.2 stating that each country should aim for a neonatal mortality rate below 12 by 2030 [4]. Helping Babies Breathe (HBB) [5] is a basic neonatal resuscitation curriculum for low-resource settings aiming to improve skilled attendance at birth. National roll-out of HBB has been one of the cornerstones to further reduce the neonatal mortality [6,7].

Although the neonate´s transition from oxygenation from the placenta to pulmonary-based oxygenation is an intricate orchestration involving many aspects, for most neonates the process is uncomplicated [8]. Of all neonates born in the world annually, around 5-6 percent (7-9 million) will need neonatal resuscitation at birth [9]. High quality resuscitation has the potential to prevent neonatal deaths and complications from birth asphyxia. Positive-pressure ventilation (PPV) to ensure inflation of lungs and onset of alveolar aeration is key to successful resuscitation [10]. According to international guidelines, PPV should be initiated within one minute from birth and heart rate assessed after 30-60 seconds [11,12].

Video recordings have been suggested to be a valuable tool to evaluate resuscitation performance during neonatal resuscitations [13,14]. Difficulties to properly perform PPV have been reported even when highly experienced neonatologists resuscitate neonates [15,16]. In low-resource settings, clinical practices of neonatal resuscitation have rarely been examined. In this prospective observational study, we aimed to detail the crucial events of neonatal resuscitation performed by HBB-trained birth attendants in a low-resource setting. Our primary objective was to investigate the cumulative proportion of ventilation time during the first minute in neonates in need of PPV. Secondary objectives included examining interruptions in PPV and the cause for the interruptions.

METHODS

Study design

This observational study was performed utilizing video recordings of neonatal resuscitations at Mulago National Referral Hospital from 10th November 2015 to 26th January 2016. The sample was limited to the neonatal resuscitation attempts recorded during the study period for practical reasons, and given similar studies, a sample size of 140 resuscitation attempts were considered acceptable. Inclusion criteria were neonates needing PPV support taken to the resuscitation table where birth attendants, HBB trained or certified by similar neonatal resuscitation programmes, initiated the resuscitation. Exclusion criteria were recordings of resuscitations with bad video quality, recordings where no resuscitation was done, recordings were there were no apparent signs of life after resuscitation, resuscitations using laryngeal

Confidential: For Review Only

mask airway and resuscitations of neonates weighing ≤1000 grams. We also excluded the resuscitations where advanced resuscitation practices were initiated immediately upon arrival on table. Cases missing a report form or a video recording were also excluded.

Study setting

The Mulago National Referral Hospital is located in the capital city Kampala. The study was conducted at the high-risk labour ward and operating theatre, Department of Obstetrics and Gynaecology. The labour ward had 16 beds with one resuscitation table, and approximately 40-60 vaginal or assisted deliveries daily. On average, 15-20 caesarean sections were carried out per day in the two theatres, each with a neonatal resuscitation table. All three resuscitation tables were equipped with basic resuscitation equipment including 250 ml self-inflating bags and masks of size 0 and 1 for PPV as well as nasal cannulas with oxygen and bulb syringes for suction. Facemask was the most used tool used for PPV, while laryngeal mask airway was sometimes used.

Patient and public involvement

It was deemed not appropriate to involve patients or the public in the design, or conduct, or reporting or dissemination plans for the study.

Study procedures and data collection

All neonates needing PPV placed on the open resuscitation table during the study period were video recorded and assessed for eligibility. The research team estimated the time from birth to arrival to the resuscitation table to a mean time of 90 seconds in the labour ward and 30 seconds in theatre after observations and discussions with local birth attendants and hospital officials. The cameras (D-Link DCS 820L) were equipped with motion detection and started recording when a neonate was brought to the resuscitation table. Only the neonate and hands of the birth attendants were visible. The data from the cameras were downloaded and stored on securely kept external hard drives.

The free-of-charge iPad application NeoTapAS (Advanced Support) [17] was used to log and time the various events observed in the video recordings. There are two versions one for smartphones (NeoTapLS) and one for tablets (NeoTapAS). Studies have shown NeoTap to be a reliable, accurate and fast aid in assessing neonatal resuscitations [18–21]. Two investigators (DH and DM) reviewed and logged times and events for each included resuscitation. This included the duration of time of PPV, stimulation and suction. An interruption in ventilation was any type of activity that was not PPV after the PPV had started, for example stimulation or suction. A suction event was defined as when the bulb syringe was inserted in a nostril or the mouth and then removed. The head and mask position during PPV were observed, and if not the recommended neutral or sniffing position, or accurate mask placement described in the HBB were present this was noted together with if any type of heart assessment were made. If discrepancies arouse between the two logs, the first investigator (DH) reviewed the

Confidential: For Review Only

resuscitation again and made a final decision. This occurred for 25 resuscitations. From the NeoTapAS application the data were transferred to R [22] for statistical analysis.

Written and informed consent was obtained from birth attendants working at the labour ward and theatre during the study period. Qualifications, neonatal resuscitation training and whether they used the HBB action plan during their daily practice were noted. Deferred consent was obtained from the mothers of neonates being resuscitated before storing or viewing the corresponding video recording. A case report form containing the neonate's time of birth, mode of delivery, who delivered the baby, birth weight, gender, birth type (singleton or twin), Apgar score at 1 and 5 minutes, any abnormal features, the mother's socio-demographics, antenatal and natal history was filled in after the resuscitation if deferred consent was obtained.

Statistical analysis

Descriptive information regarding study selection process is presented along with descriptive statistics of mothers, neonates and the birth attendants in numbers (%). Due to the different locations and modes of birth, resuscitations taking place in the labour ward were compared with resuscitations taking place in theatre. The primary and secondary outcomes were analysed through descriptive statistics with numbers (%) for categorial variables and median (interquartile range) and compared through Fisher's exact test and Wilcoxon rank sum test due to the dependent variable having low number of counts and being non-parametric. All quantitative data were analysed in R [22]. A p-value <0.05 was considered to be statistically significant.

RESULTS

In total 141 resuscitation attempts were recorded, of these 42 were excluded primarily due to use of laryngeal mask airway and no consent or case report form, which resulted in 99 video recordings eligible for timing and logging (**Figure 1**).

Baseline characteristics

Pregnancy complications were common (**Table 1**). Vaginal and caesarean delivery mode ratio was 1:1. The median number of neonates with Apgar score 0-3 at minute 1 and minute 5 was 28 (28%) and 6 (6%), respectively. A shift in overall median Apgar score between first and fifth minute from 4-6 to ≥7 was observed. Most neonates weighed ≥3000 grams. All birth attendants had at least a midwifery certificate and all but 4 birth attendants claimed to use the HBB action plan during resuscitations.

Confidential: For Review Only

Table 1. Characteristics of included mothers, neonates and birth attendants.

**Mothers**	Number N = 99 n (%)	**Neonates**	Number N = 99 n (%)	**Birth attendants**	Number N = 45 n (%)
**Age (years)**		**Gender**		**Age (years)**	
15 – 19	15 (15)	Female	52 (52)	20 – 30	14 (31)
20 - 24	42 (42)	Male	47 (47)	31 – 41	21 (47)
30 – 34	10 (10)	**Birth type**		42 – 52	10 (23)
35 – 49	11 (11)	Singleton	90 (90)	**Qualification level**	
**Antenatal attendance**		Twins	9 (9)	Degree nurse	2 (4)
Yes	96 (97)	**Apgar score at 1 minute**		Diploma midwife	27 (60)
No	3 (3)	0 – 3	28 (28)	Certificate midwife	16 (36)
**Birth order**		4 – 6	58 (58)	**Attended HBB refresher course**	
First child	38 (38)	≥ 7	12 (12)	Yes	27 (60)
Second child	19 (19)	**Apgar score at 5 minutes[2]**		No	18 (40)
≥ Third child	42 (42)	0 – 3	6 (6)	**Time since last HBB refresher course**	
**Pregnancy complications**		4 – 6	45 (45)	≤ 6 months	10 (37)
Obstructed labour	14 (14)	≥ 7	48 (48)	6 – 12 months	4 (15)
Fetal distress	4 (4)	**Birth weight in grams**		≥ 12 months	13 (48)
Pre-eclampsia/eclampsia	3 (3)	1000 – 1499	7 (7)	**Reasons for not attending HBB refresher course[3]**	
Breech	5 (5)	1500 – 1999	9 (9)	I have adequate knowledge	8 (18)
Cord prolapse	2 (2)	2000 – 2999	26 (26)	No time	9 (20)
Oligohydramnios	6 (6)	≥ 3000	56 (56)	No finances	10 (22)
Preterm birth	1 (1)			No such course was offered	4 (9)
Other[1]	16 (16)			No opportunity	5 (11)
No complications	48 (48)			No answer	2 (4)
**Mode of delivery**				**Used the HBB action plan during resuscitations**	
Spontaneous vaginal delivery	48 (48)			Yes	41 (91)
Caesarean section	48 (48)			No	4 (9)
Instrumental delivery	3 (3)				

[1]Other: cervical dystocia, bleeding conditions such as placenta previa or placenta abruption, delayed second stage of labour which in 14 out of 16 occurrences resulted in a caesarean section. [2]Apgar score at 10 minutes was not included as most attention is given to the scoring at 5 minutes.

Ventilation

The median (IQR) time from birth to initiation of PPV was 137 (94-168) seconds, and from arrival to table 66 (44-102) seconds (**Table 2**). There was minimal PPV during minute 1 after arrival to the resuscitation table. In resuscitations taking place in the labour ward there were 0 (0-10) seconds of PPV and 0 (0-12) seconds in the theatre, and during minute 2 resuscitations taking place in the labour ward included 19 (7-32) seconds of PPV while resuscitations in the theatre had 18 (8-25) seconds of ventilation. Overall, during the first 2 minutes after arriving on the table there had only been 21 (12-36) seconds of PPV with no significant difference between the place of birth. Additionally, approximately a third of the

Confidential: For Review Only

neonates did not have the recommended neutral to sniffing head position during ventilation and the resuscitation was mainly performed by a single birth attendant. The duration of interruptions in ventilations during minute 1 after initiating PPV for all resuscitations was 25 (15-35) and 29 (24-38) seconds of interruption, respectively, **Figure 2** and **Table 2**. Interruptions persisted, and for the first 2 minutes after initiating PPV there were 72 (47-89) seconds of interruption for both groups. Stimulation 28% (n=67), no apparent reason 26% (n=61) and suction 22% (n=51) were the primary causes for interruptions, while corrective adjustments of the position of the neonate and change of facemask were also common causes (**Figure 3**).

Table 2. Resuscitation procedures during first and second minute after arriving at the resuscitation table.

**Place of birth**	Total (N= 99)	Labour ward (N= 51)	Theatre (N=48)	p-value
**Time**	**Seconds (IQR)**	**Seconds (IQR)**	**Seconds (IQR)**	
**Ventilation**				
Time to initiation of PPV from birth[1]	137 (94-168)	161 (142-197)	92 (68-113)	<0.001
Time to initiation of PPV after arrival on table	66 (44-102)	71 (52-107)	62 (38-83)	0.126
Total duration of PPV minute 1	0 (0-10)	0 (0-10)	0 (0-12)	0.159
Total duration of PPV minute 2	18 (7-30)	19 (7-32)	18 (8-25)	0.623
Total duration of PPV first 2 minutes	21 (12-36)	19 (9-43)	23 (14-32)	0.616
Total duration of interruption minute 1 of ventilation	28 (18-37)	25 (15-35)	29 (24-38)	0.132
Total duration of interruption first 2 minutes of ventilation	72 (47-89)	68 (45-84)	81 (57-93)	0.059
**Stimulation**				
Total stimulation time minute 1	9 (3-18)	5 (0-9)	16 (11-21)	<0.001
Total stimulation time minute 2	5 (0-10)	4 (0-8)	2 (6-10)	0.125
Total stimulation time first 2 minutes	16 (7-26)	8 (4-21)	24 (15-29)	<0.001
**Suction**				
Total suction time minute 1	21 (8-29)	23 (10-33)	18 (6-25)	0.136
Total suction time minute 2	7 (0-20)	4 (0-21)	9 (0-19)	0.244
Total suction time first 2 minutes	28 (15-43)	28 (16-44)	29 (14-43)	0.737
**Frequency**	**Number (%)**	**Number (%)**	**Number (%)**	
Only one birth attendant	62 (63)	29 (57)	33 (69)	0.299
**Ventilation procedure**				
Not recommended head position	31 (31)	8 (16)	23 (48)	0.001
Not accurate mask position	16 (16)	5 (10)	11 (23)	0.103
**Heart rate assessment**				
By auscultation	1 (1)	1 (2)	0 (0)	1
By umbilical cord palpation	5 (5)	1 (2)	4 (8)	0.196
By chest palpation	11 (11)	9 (18)	2 (4)	0.052

Results are presented as median seconds (IQR) or numbers (%) and compared with Wilcoxon rank sum test for continuous and Fisher's exact test for categorical variables. [1] For resuscitations taking

Confidential: For Review Only

place at the labour ward (n=51) and theatre (n=48) the estimated time from birth to resuscitation was 90 seconds and 30 seconds respectively. All other measurements in the table are from when the neonate arrives on the resuscitation table.

Stimulation and suction

Stimulation time during minute 1 was significantly lower for the resuscitations taking place in the labour ward 5 (0-9) versus 16 (11-21) seconds (p-value <0.001), stimulation was less present in minute 2 (**Table 2**). Overall, for the first 2 minutes after arriving to the table the duration of stimulation was 16 (7-26) seconds. Suction was prevalent for the whole resuscitation but particularly during minute 1, the cumulative suction time for both groups was 21 (8-29) seconds with a median of 5 (2-8) suction events with bulb syringes per neonate. During minute 2 there was less duration of suction in both groups. A median of 8 (4-13) separate suction events per neonate which led to a cumulative time effort of 28 (15-43) seconds were recorded during the first 2 minutes after arriving to the resuscitation table. Lastly, only 17% of the resuscitations included any form of heart rate assessment.

DISCUSSION

This observational study used video recordings of neonatal resuscitations in a country with high neonatal mortality. Our findings demonstrate a low adherence to standard resuscitation practices in 2015-2016 with inadequate initiation and duration of PPV, tendency to overstimulate, and excessive focus on suction regardless of mode and place of birth. This adds to the comparable resuscitation patterns with insufficient or incorrect ventilation efforts found in a similar setting in Mozambique [23], while a study in Nepal concluded that none of the examined resuscitations met the golden minute guideline standard [24]. However, failing to initiate proper ventilation within the first minute and applying suboptimal PPV is also common in high-resource settings [25–27]. Specifically, a study from a tertiary hospital in Norway report a median time from arriving to the table to initiation of PPV of 42 seconds and 56% of neonates received PPV with a 60% ventilation fraction during the first 30 seconds. [25] The time to initiation of PPV was considerably longer in our study and does not commence within the recommended golden minute after birth. However, primarily due to logistical reasons, the time from birth to resuscitation was significantly longer for neonates in the labour ward. In general it seems that ventilation during resuscitations tend not to be commenced within the recommended time in both high- and low-resource settings.

In our study, the high number and relatively long duration of interruptions was reflected in the low ventilation time during the first minute of ventilation not meeting the recommended 60 seconds of continuous PPV before assessing the adequacy of ventilation, with no signifcant difference between resuscitations taking place in the labour ward and theatre. We build on the previous literature by showcasing what the actual causes of those interruptions were. In our study, most interruptions were due to stimulation, unknown reasons, and suctioning, which should not disrupt PPV. In the Norwegian study [25] the main reasons for ventilation interruptions were for adjustments for optimising ventilation, heart rate evaluation, and

Confidential: For Review Only

stimulation. This was often not the case in our study. It is, however, important to acknowledge and stress the difference in staff quantity and resources between these two settings. According to a qualitative study from Tanzania the main reasons, as per the birth attendants themselves, for delay or interruptions of PPV were fear of doing a poor job in an acute situation, difficulties in assessing the neonate and in taking appropriate action [28]. Similar findings have been reported from high-resource settings as well [29]. It is highly likely that the lack of adherence to guidelines in our study stems from both a lack of knowledge and professional confidence, compounded by the limited resources and low number of staff.

Our study reveals excessive amounts of both stimulation and suctioning, the former being the main reason for ventilation interruptions adding strength to similar findings in other settings [25,30,31]. In particular, neonates in need of resuscitation in the theatre were stimulated for a longer duration than those in the labour ward, in part the discrepancy could be caused by a difference in perception of the need for stimulation after birth depending on the mode of delivery. Intriguingly, Wrammert et al. [32] showed excessive usage of both stimulation and suctioning as main reasons to ventilation delay and halt in resuscitations conducted in Nepal, and proposed that the continuation of the phenomenon was due to difficulties of abandoning a tradition of suctioning of non-breathing neonates before the implementation of HBB or similar educational programs. Further, it is understandable that in an acute situation where time is of the essence, one might want to utilize other strategies if ventilation seems fruitless. Excessive usage of stimulation and suctioning in our study could be explained by this need, requiring special attention when developing educational programs. Lastly, heart rate assessments were rarely done, a vital component of neonatal resuscitation often underused in low-resource settings [33].

Almost all birth attendants claimed to be using the HBB action plan routinely when working with neonatal resuscitation. The results in our study however display a real-world situation where set guidelines were not followed. The need for and beneficial effects of acquired skills and knowledge to be frequently updated, practiced and evaluated is clear while similarly important is to increase staff numbers and forming close working professional teams for resuscitations [24,34–36].

Strengths and weaknesses

Most limitations of this study are inherent to post-hoc video analysis. First, the factual time spent between birth and arrival to table was estimated and may not reflect the correct time in each case. Second, assessing recorded resuscitation procedures relies on the clinical expertise of the reviewer, and distinguishing between different procedures is not always clear. Third, though an improvement in Apgar scores from minute 1 to minute 5 was observed, it was not possible to evaluate the effectiveness of PPV since no certain observation of tidal volumes or pulse oximetry was performed. However, by using video recordings the most critical aspects of actual resuscitation practices can be examined and

Confidential: For Review Only

timely logged, and an overall picture formed.

Conclusion

This study demonstrates real life neonatal resuscitation practices in 2015-2016 that are not in line with guidelines with inadequate initiation and duration of PPV, tendency for overstimulation and excessive focus on suction. There is a strong need for continuous educational efforts and investments in adequate resources to increase the quality of clinical neonatal resuscitation practices in low-resource settings.

Acknowledgements

We would like to give thanks to all participants of this study. Our greatest thanks to the staff at the Maternity and Special Care Unit of Mulago National Referral Hospital, Kampala, Uganda, for their continuous struggle to improve the care of neonates.

Contributors

DH was responsible for data analysis, interpretation of data and for the writing process including writing a first draft of the manuscript and approval of the final draft. SMH was responsible for the study design and acquisition of data from video recordings. She revised the manuscript and approved the final draft. NJP contributed to study design and acquisition of data. He revised the manuscript and approved the final draft. DM assisted in data analysis and writing process, revised the manuscript and approved the final draft. CL contributed to study design, was responsible for preparation of the study site and collecting the video recordings. She revised the manuscript and approved the final draft. TT contributed to study design, interpretation of data, manuscript revision and approved the final draft. TA contributed to the study design, data analysis, manuscript revision and approval of the final draft.

Funding

The study was funded by the Norwegian Government through the Norwegian Quota Scholarship scheme and by in-kind contributions from the University of Bergen, Norway, Makerere University, Uganda, and the Karolinska Institutet, Sweden.

Competing interests

The authors have no financial relationships relevant to this article to disclose. SMH, NJP, CL, TT and TA are co-founders of the non-profit organisation Tap4Life (www.tap4life.org), which produced the free-of-charge application NeoTap. The SMH, NJP, CL, TT and TA do not get any salary from Tap4Life.

Confidential: For Review Only

Patient consent for publication

Not required.

Ethical approval

Ethical approval of the study was received from the Mulago Hospital Research and Ethics Committee and the Uganda National Council of Science and Technology (MREC:841). The Norwegian research ethics committee exempted it from evaluation since it was deemed a quality assurance study.

Data availability statement

All data relevant to this study are included in the article.

References

1 Ehret DY, Patterson JK, Bose CL. Improving Neonatal Care: A Global Perspective. Clin. Perinatol. 2017;**44**:567–82. doi:10.1016/j.clp.2017.05.002

2 Liu L, Oza S, Hogan D, *et al.* Global, regional, and national causes of child mortality in 2000-13, with projections to inform post-2015 priorities: an updated systematic analysis. *Lancet* 2015;**385**:430–40. doi:10.1016/S0140-6736(14)61698-6

3 UN Inter-agency Group for Child Mortality Estimation. Levels & Trend in Child Mortality: Report 2020. New York: 2020. https://www.unicef.org/media/79371/file/UN-IGME-child-mortality-report-2020.pdf.pdf

4 Hug L, Alexander M, You D, *et al.* National, regional, and global levels and trends in neonatal mortality between 1990 and 2017, with scenario-based projections to 2030: a systematic analysis. *Lancet Glob Heal* 2019;**7**:e710–20. doi:10.1016/S2214-109X(19)30163-9

5 Kamath-Rayne BD, Thukral A, Visick MK, *et al.* Helping Babies Breathe, second edition: A model for strengthening educational programs to increase global newborn survival. *Glob Heal Sci Pract* 2018;**6**:538–51. doi:10.9745/GHSP-D-18-00147

6 Reisman J, Arlington L, Jensen L, *et al.* Newborn resuscitation training in resource-limited settings: A systematic literature review. Pediatrics. 2016;**138**. doi:10.1542/peds.2015-4490

7 Kamath-Rayne BD, Berkelhamer SK, Kc A, *et al.* Neonatal resuscitation in global health settings: An examination of the past to prepare for the future. *Pediatr Res* 2017;**82**:194–200. doi:10.1038/pr.2017.48

8 Morton SU, Brodsky D. Fetal Physiology and the Transition to Extrauterine Life. Clin. Perinatol. 2016;**43**:395–407. doi:10.1016/j.clp.2016.04.001

9 UNICEF. State of the World's Children 2019: Children, food and nutrition. New York: 2019. https://www.unicef.org/reports/state-of-worlds-children-2019

Confidential: For Review Only

10 Hooper SB, Siew ML, Kitchen MJ, *et al.* Establishing functional residual capacity in the non-breathing infant. Semin. Fetal Neonatal Med. 2013;**18**:336–43. doi:10.1016/j.siny.2013.08.011

11 Perlman JM, Wyllie J, Kattwinkel J, *et al.* Part 7: Neonatal resuscitation 2015 international consensus on cardiopulmonary resuscitation and emergency cardiovascular care science with treatment recommendations. *Pediatrics* 2015;**136**:S120–66. doi:10.1542/peds.2015-3373D

12 World Health Organization. Guidlines on basic newborn resucitation. Geneva: 2012. https://apps.who.int/iris/bitstream/handle/10665/75157/9789241503693_eng.pdf?sequence=1

13 Carbine DN, Finer NN, Knodel E, *et al.* Video Recording as a Means of Evaluating Neonatal Resuscitation Performance. *Pediatrics* 2000;**106**:654–8. doi:doi: 10.1542/peds.106.4.654

14 Schilleman K, Siew ML, Lopriore E, *et al.* Auditing resuscitation of preterm infants at birth by recording video and physiological parameters. *Resuscitation* 2012;**83**:1135–9. doi:10.1016/j.resuscitation.2012.01.036

15 Schmölzer GM, Dawson JA, Kamlin COF, *et al.* Airway obstruction and gas leak during mask ventilation of preterm infants in the delivery room. *Arch Dis Child Fetal Neonatal Ed* 2011;**96**:F254-7. doi:10.1136/adc.2010.191171

16 Moshiro R, Perlman JM, Kidanto H, *et al.* Predictors of death including quality of positive pressure ventilation during newborn resuscitation and the relationship to outcome at seven days in a rural Tanzanian hospital. *PLoS One* 2018;**13**:e0202641. doi:10.1371/journal.pone.0202641

17 Tap4Life. http://tap4life.org/

18 Myrnerts Höök S, Pejovic NJ, Marrone G, *et al.* Accurate and fast neonatal heart rate assessment with a smartphone-based application – a manikin study. *Acta Paediatr Int J Paediatr* 2018;**107**:1548–54. doi:10.1111/apa.14350

19 Johnson PA, Morina N, O'Reilly M, *et al.* Evaluation of a Tap-Based Smartphone App for Heart Rate Assessment During Asphyxia in a Porcine Model of Neonatal Resuscitation. *Front Pediatr* 2019;**7**:453. doi:10.3389/fped.2019.00453

20 Binotti M, Cavallin F, Ingrassia PL, *et al.* Heart rate assessment using NeoTapAdvancedSupport: A simulation study. *Arch Dis Child Fetal Neonatal Ed* 2019;**104**:F440–2. doi:10.1136/archdischild-2018-315408

21 Myrnerts Höök S, Pejovic NJ, Cavallin F, *et al.* Smartphone app for neonatal heart rate assessment: an observational study. *BMJ Paediatr Open* 2020;**4**:e000688. doi:10.1136/bmjpo-2020-000688

22 R Core Team. R: A language and environment for statistical computing. 2019.https://www.r-project.org/

Confidential: For Review Only

23 Trevisanuto D, Bertuola F, Lanzoni P, *et al.* Effect of a neonatal resuscitation course on healthcare providers' performances assessed by video recording in a low-resource setting. *PLoS One* 2015;**10**:1–12. doi:10.1371/journal.pone.0144443

24 Kc A, Wrammert J, Clark RB, *et al.* Reducing perinatal mortality in Nepal using helping babies breathe. *Pediatrics* 2016;**137**:e20150117. doi:10.1542/peds.2015-0117

25 Skåre C, Boldingh AM, Nakstad B, *et al.* Ventilation fraction during the first 30 s of neonatal resuscitation. *Resuscitation* 2016;**107**:25–30. doi:10.1016/j.resuscitation.2016.07.231

26 Bjorland PA, Ersdal HL, Øymar K, *et al.* Compliance with guidelines and efficacy of heart rate monitoring during newborn resuscitation: A prospective video study. *Neonatology* 2020;**117**:175–81. doi:10.1159/000506772

27 Niles DE, Cines C, Insley E, *et al.* Incidence and characteristics of positive pressure ventilation delivered to newborns in a US tertiary academic hospital. *Resuscitation* 2017;**115**:102–9. doi:10.1016/j.resuscitation.2017.03.035

28 Moshiro R, Ersdal HL, Mdoe P, *et al.* Factors affecting effective ventilation during newborn resuscitation: a qualitative study among midwives in rural Tanzania. *Glob Health Action* 2018;**11**:1423862. doi:10.1080/16549716.2018.1423862

29 Ljungblad LW, Skovdahl K, McCormack B, *et al.* Balancing life and death during the golden minute – midwives' experiences of performing newborn resuscitation. *J Multidiscip Healthc* 2020;**13**:943–52. doi:10.2147/JMDH.S268959

30 Bansal SC, Nimbalkar AS, Patel D V., *et al.* Current Neonatal Resuscitation Practices among Paediatricians in Gujarat, India. *Int J Pediatr* 2014;**2014**:1–7. doi:10.1155/2014/676374

31 Adams E, O´Neal P, Hanson L, *et al.* Airway clearance of the term newborn. *MCN Am J Matern Child Nurs* 2018;**43**:306–12. doi:10.1097/NMC.0000000000000473

32 Wrammert J, Zetterlund C, Ashish KC, *et al.* Resuscitation practices of low and normal birth weight infants in Nepal: An observational study using video camera recordings. *Glob Health Action* 2017;**10**:1322372. doi:10.1080/16549716.2017.1322372

33 Johnson PA, Schmölzer GM. Heart Rate Assessment during Neonatal Resuscitation. *Healthcare* 2020;**8**:43. doi:10.3390/healthcare8010043

34 Hodgins S. Helping babies breathe - beyond training. *Glob Heal Sci Pr* 2018;**6**:402–4. doi:10.1016/j.jpeds.2012.03.030

35 Bang A, Patel A, Bellad R, *et al.* Helping Babies Breathe (HBB) training: What happens to knowledge and skills over time? *BMC Pregnancy Childbirth* 2016;**16**:1–12. doi:10.1186/s12884-016-1141-3

36 Seto TL, Tabangin ME, Josyula S, *et al.* Educational outcomes of Helping Babies Breathe training at a community hospital in Honduras. *Perspect Med Educ* 2015;**4**:225–32. doi:10.1007/s40037-015-0214-8

Confidential: For Review Only

Figure legends

Figure 1. Study profile.

Figure 2. The duration of ventilation and interruptions in ventilation (seconds) during first minute after initiating positive pressure ventilation in the 99 resuscitations.

Figure 3. Cause of interruptions during first 2 minutes of initiated positive pressure ventilation.

Confidential: For Review Only

Confidential: For Review Only

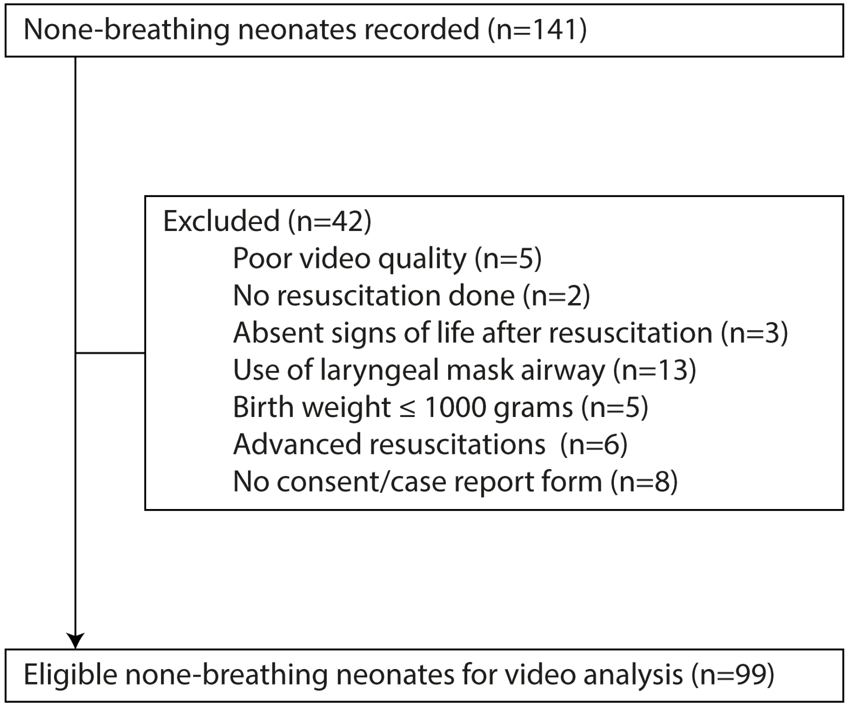
None-breathing neonates recorded (n=141)
Excluded (n=42)
Poor video quality (n=5)
No resuscitation done (n=2)
Absent signs of life after resuscitation (n=3)
Use of laryngeal mask airway (n=13)
Birth weight ≤ 1000 grams (n=5)
Advanced resuscitations (n=6)
No consent/case report form (n=8)
Eligible none-breathing neonates for video analysis (n=99)

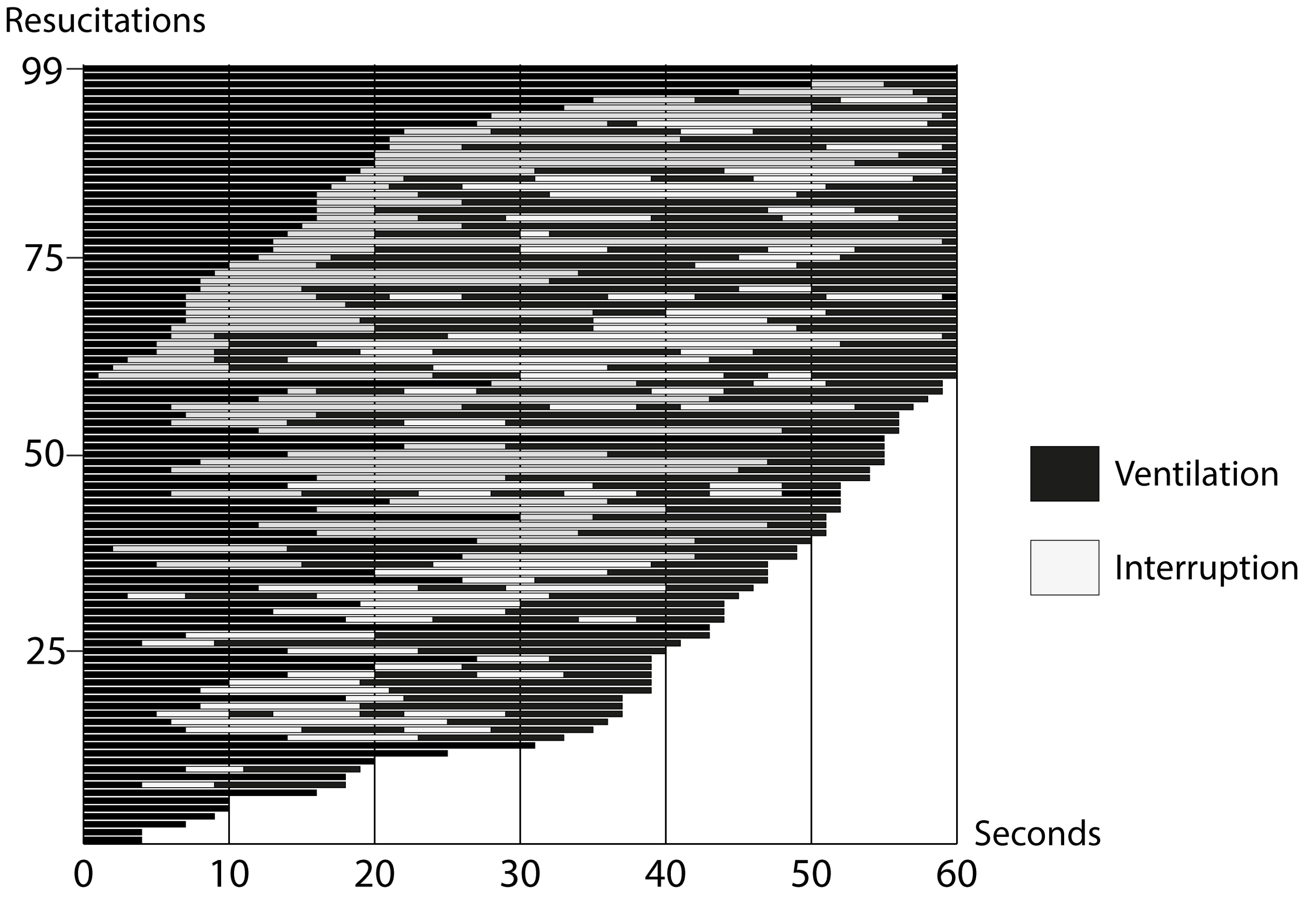
Resucitations
99
75
50
25
0
10
20
30
40
50
60
Seconds
Ventilation
Interruption

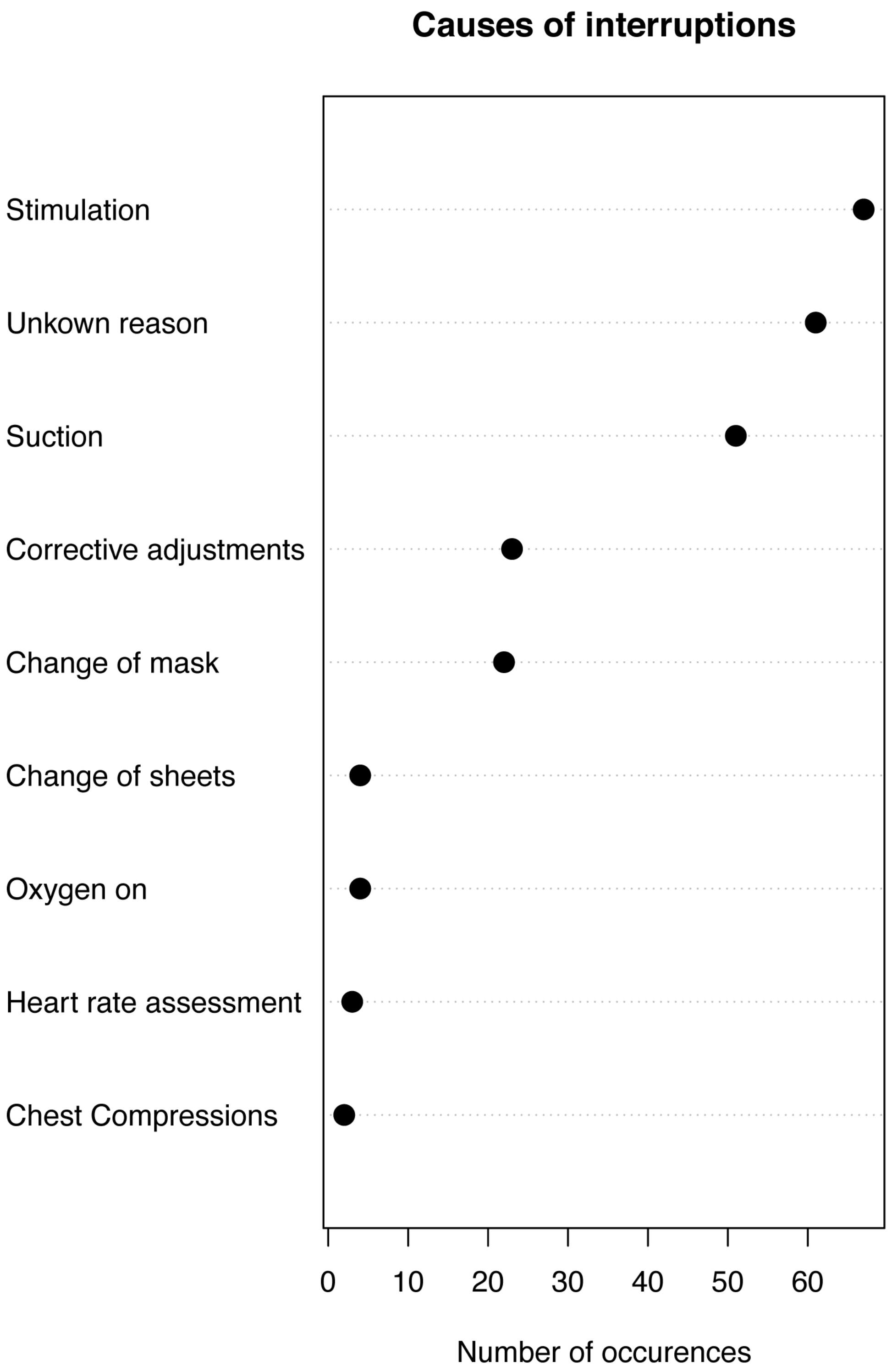
Causes of interruptions
Stimulation
Unkown reason
Suction
Corrective adjustments
Change of mask
Change of sheets
Oxygen on
Heart rate assessment
Chest Compressions
0
10
20
30
40
50
60
Number of occurences
